# Synthesis, crystal structure, and properties of methyl-substituted coronene amide analogue†

**DOI:** 10.1039/d2ra04035b

**Published:** 2022-09-16

**Authors:** Kenta Rakumitsu, Miho Fujii, Sotaro Kusumoto, Shoko Kikkawa, Isao Azumaya, Akihiro Yokoyama

**Affiliations:** Faculty of Science and Technology, Seikei University 3-3-1 Kichijoji-kitamachi, Musashino Tokyo 180-8633 Japan ayokoyama@st.seikei.ac.jp; Department of Material and Life Chemistry, Faculty of Engineering, Kanagawa University 3-27-1 Rokkakubashi, Kanagawa-ku Yokohama 221-8686 Japan; Faculty of Pharmaceutical Sciences, Toho University 2-2-1 Miyama, Funabashi Chiba 274-8510 Japan

## Abstract

A coronene amide analogue was synthesized in six steps using an improved method at the final biarylation step. The key to the progress of palladium-mediated biarylation involved the introduction of three methyl groups to suppress the undesired reaction and the use of tri-*tert*-butylphosphine as the ligand for palladium. Single-crystal X-ray analysis revealed that the core unit of the coronene analogue has a non-planar structure.

## Abstract 2

The palladium-mediated intramolecular biarylation of the cyclic triamide of 2-bromo-4-(isobutylamino)benzoic acid proceeded effectively by the introduction of three methyl groups and the use of tri-*tert*-butylphosphine as the ligand.

## Graphical abstract

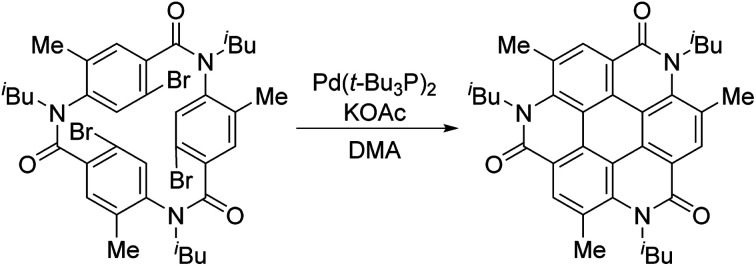

## Introduction

**Fig. 1:**
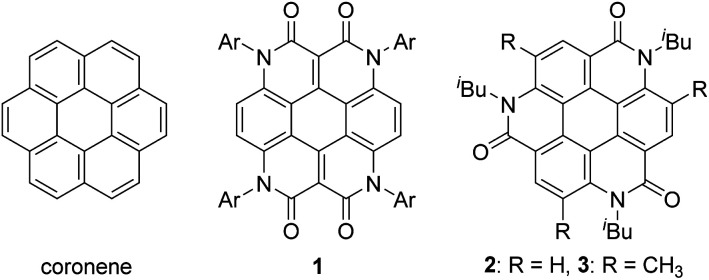
Coronene and its amide analogues 1–3.

**Scheme 1:**
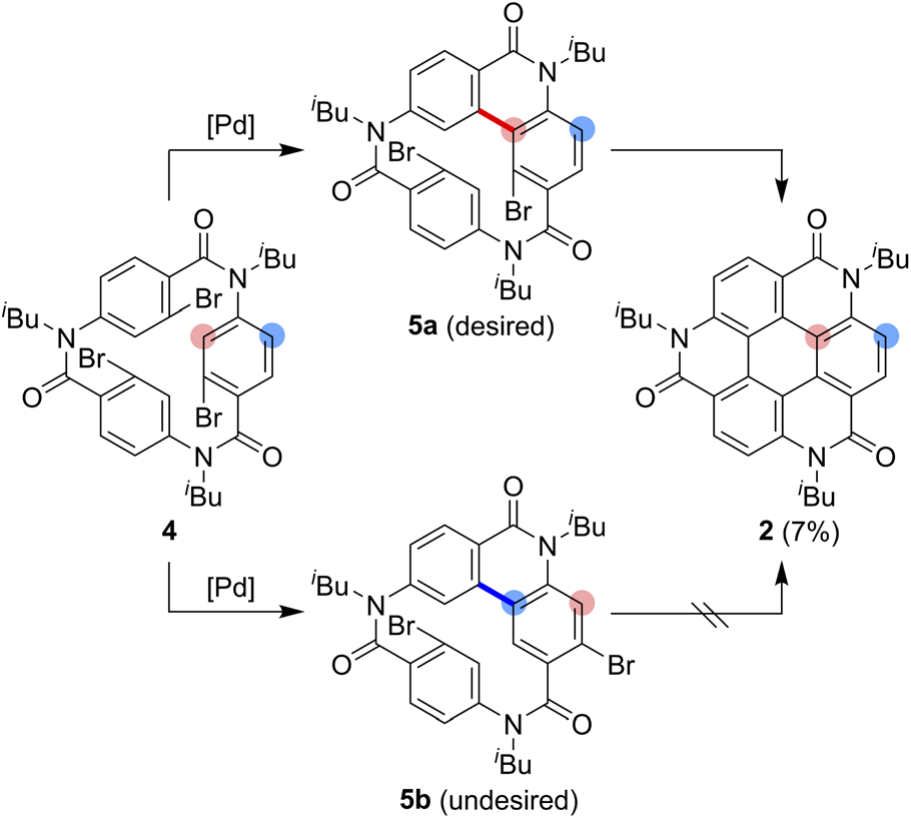
Synthesis of the coronene analogue 2.

**Scheme 2:**
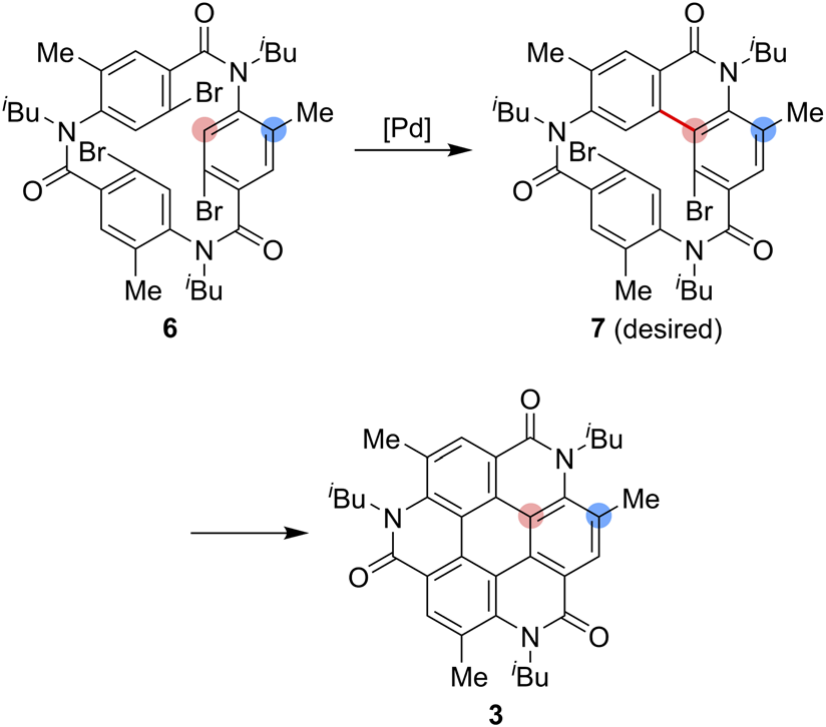
Synthesis of the coronene analogue 3.

As one of the most basic polycyclic aromatic hydrocarbons, coronene comprises one central benzene ring and six surrounding benzene rings (Fig. 1). It is characterized by *D*_6h_ symmetry and a completely localized π-conjugated system.^1^ The size of coronene is suitable for processing techniques. Several applications have been investigated, including building blocks for self-assembling compounds^2–4^ and organic transistors.^5–8^ Doping of heteroatoms in aromatic rings is an effective approach for modulating physico-electrochemical properties, and several synthetic studies of heterocoronenes have been reported.^9–20^ As a coronene analogue containing amide linkages, the synthesis^17^ and application to organic semiconductors^18^ of a *D*_2h_ symmetric compound 1 have been reported. Recently, our group has synthesized 2, a new *C*_3_ symmetrical coronene analogue with three peripheral amide linkages, by the cyclic trimerization of 2-bromo-4-(isobutylamino)benzoic acid, followed by palladium-mediated intramolecular biarylation.^21^ The novelty of our procedure is the construction of peripheral bonds of coronene analogue first, followed by the formation of central benzene unit. However, the yield of 2 was low (7%) due to biarylation between the undesired positions. That is, as shown in Scheme 1, the intramolecular biarylation of 4 between the bromo-substituted carbon atom and the neighboring aromatic carbon atom designated by red color afforded 5a, and the repetition of the same reaction afforded 2. In contrast, the intramolecular biarylation at the carbon atom designated by blue afforded 5b, which can no longer be transformed into 2. To explore the applications of coronene amide analogues, more efficient synthetic protocol should be developed. We expected that the introduction of methyl group to the 5-position of 2-bromo-4-(isobutylamino)benzoic acid unit would suppress the undesired reaction of the palladium-mediated arylation to improve the yield of the coronene amide analogue. Herein, we report the synthesis of coronene amide analogue 3 by using 6, where three methyl groups were introduced into the undesired reaction sites (Scheme 2). In addition, the crystal structure and optical properties of 3 were also reported.

## Results and discussion

**Scheme 3:**
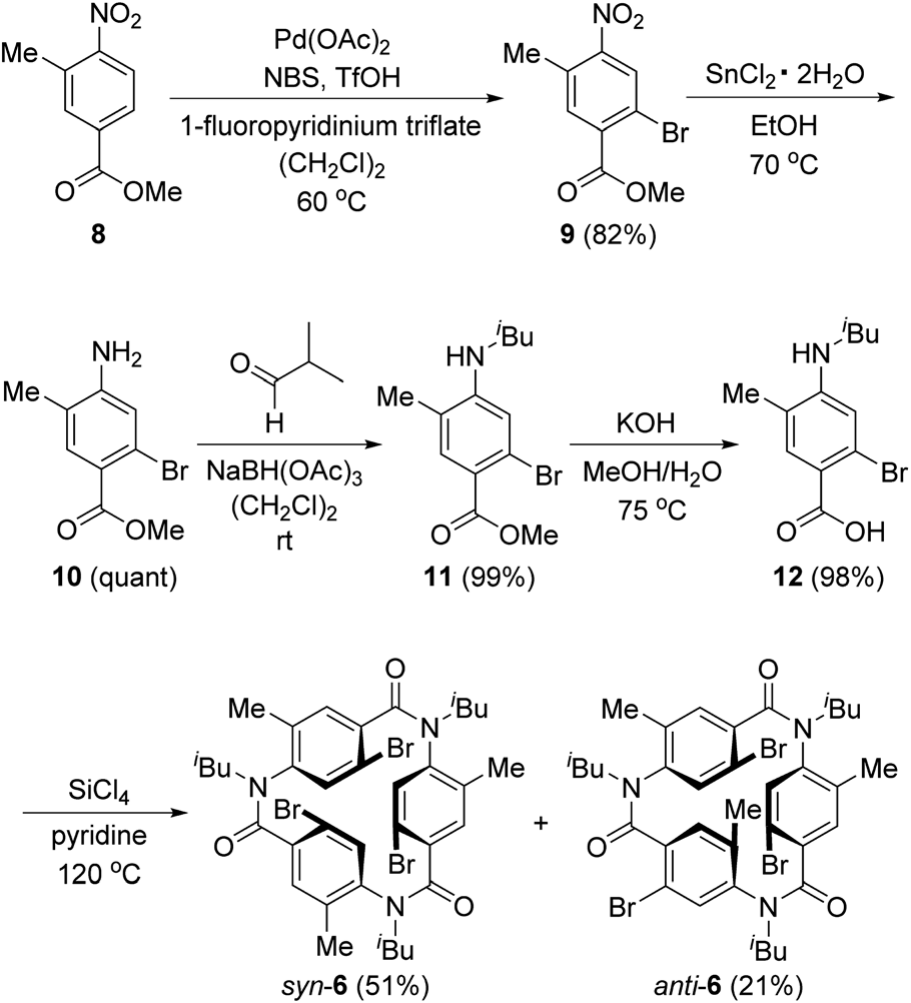
Synthesis of 6.

Scheme 3 shows the synthesis of 6. Four-substituted benzene derivative 9 was synthesized by the regioselective bromination of commercially available methyl 3-methyl-4-nitrobenzoate (8) using palladium(ii) acetate (Pd(OAc)_2_), *N*-bromosuccinimide (NBS), 1-fluoropyridinium triflate, and trifluoromethanesulfonic acid (TfOH) according to a previous study.^22^ Cyclic triamide 6 was expected to be synthesized from 9 by employing our previous method.^21^ To our delight, the introduced three methyl groups do not interfere with these reactions. That is, the reduction of the nitro group of 9 using tin chloride afforded amine 10, and the reductive amination using 10 and isobutyraldehyde afforded *N*-isobutyl compound 11. Carboxylic acid 12 was synthesized by the hydrolysis of 11 with potassium hydroxide. These three reactions proceeded in extremely high yields. When 12 was treated with silicon tetrachloride in pyridine at 120 °C, *syn*-6, in which three bromo groups are located in the same direction, and *anti*-6, in which one of the three bromo groups is in the opposite direction, were obtained in 51% and 21% yields, respectively.

Having completed the synthesis of biarylation precursor 6, our interest was focused on the synthesis of 3. Initially, *syn*-6 was used as the substrate. By employing our previously reported reaction conditions of 1 equivalent of Pd(OAc)_2_, 2 equivalents of triphenylphosphine (PPh_3_), and 20 equivalents of potassium carbonate (K_2_CO_3_) in *N*,*N*-dimethylformamide (DMF), the reaction at 100 °C for 76 h did not afford desired 3. Instead, starting material 6 was recovered in 41%, accompanied with the formation of by-product 13 in 17% yield formed by two biarylation and one debromination reactions (Table 1, entry 1). Increase in the reaction temperature to the reflux temperature of DMF led to the increase in the yield of 13 (51%) and the formation of several by-products, but 3 was not obtained (entry 2). When the amounts of Pd(OAc)_2_, PPh_3_, and K_2_CO_3_ were decreased to 0.60 equivalent, 1.2 equivalents, and 12 equivalents, respectively, 6 was recovered in 38% yield, the formation of the by-products was reduced, and trace amounts of 3 were obtained (entry 3). Use of tri(*o*-tolyl)phosphine (P(*o*-tol)_3_) and silver carbonate (Ag_2_CO_3_) as the ligand and base,^23^ respectively, did not afford 3 (entry 4), but the reaction with tri-*n*-butylphosphine (*n*-Bu_3_P) as the ligand^24^ afforded 3 in 8% yield (entry 5). Next, reactions using tri-*tert*-butylphosphine as the ligand were attempted.^25^ The reaction of *syn*-6 with bis(tri-*tert*-butylphosphine)palladium(0) (Pd(*t*-Bu_3_P)_2_, 0.16 equivalent) and potassium acetate (KOAc, 12 equivalents) in *N*,*N*-dimethylacetamide (DMA) at 120 °C afforded 3 and 13 in ∼47% and 52% yields, respectively (entry 6). To suppress undesired debromination that afforded 13, the reaction temperature was decreased to 100 °C. However, the yields of 3 and 13 decreased (entry 7). In the above reactions, thrice the amount of Pd(*t*-Bu_3_P)_2_ and KOAc compared with that used in the reported procedure was used,^25^ as three reaction sites were present in 6. When the reaction was performed using 4 equivalents of KOAc, which is the same amount as that reported in the literature, the yield of 3 increased to 63%; hence, these reaction conditions are selected as the optimum ones (entry 8). Next, the reaction of *anti*-6 was attempted. Because the reactivity of *anti*-6 was less than that of *syn*-6, the reaction was performed using 0.45 equivalents of Pd(*t*-Bu_3_P)_2_ and 12 equivalents of KOAc. However, the yield of 3 was 19% and 13 was obtained in 28% yield. This result indicated that the rotation of the amide–Ar bond in 6 is suppressed in comparison with that of 4,^26^ corresponding to the substrate used in our previously reported palladium reaction without methyl groups.^21^ The detailed reaction mechanism for the conversion of *anti*-6 to 3 is unclear at this point.

**Table 1:** Palladium-mediated intramolecular biarylation of 6^a^

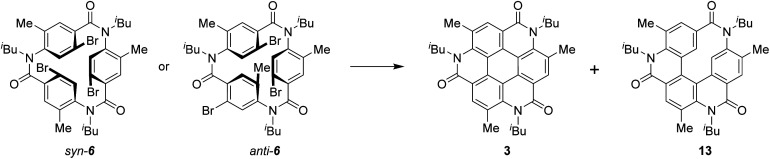										
Entry	6	Palladium (eq.)	Ligand (eq.)	Base (eq.)	Solvent	Temperature	Time (h)	Yield (%)		
								3	13	6
1	*syn*-6	Pd(OAc)_2_ (1.0)	PPh_3_ (2.0)	K_2_CO_3_ (20)	DMF	100 °C	76	0	17	41
2	*syn*-6	Pd(OAc)_2_ (1.0)	PPh_3_ (2.0)	K_2_CO_3_ (20)	DMF	reflux	74	0	51	0
3	*syn*-6	Pd(OAc)_2_ (0.60)	PPh_3_ (1.2)	K_2_CO_3_ (12)	DMF	reflux	68	trace	30	38
4	*syn*-6	Pd(OAc)_2_ (0.58)	P(*o*-tol)_3_ (2.0)	Ag_2_CO_3_ (5.8)	DMF	reflux	24	0	43	0
5	*syn*-6	Pd(OAc)_2_ (0.61)	*n*-Bu_3_P (1.9)	K_2_CO_3_ (5.9)	DMF	reflux	25	8	<25^b^	34
6	*syn*-6	Pd(*t*-Bu_3_P)_2_ (0.16)	—	KOAc (12)	DMA	120 °C	26	<47^b^	<52^b^	0
7	*syn*-6	Pd(*t*-Bu_3_P)_2_ (0.16)	—	KOAc (12)	DMA	100 °C	25	<39^b^	<34^b^	29
8	*syn*-6	Pd(*t*-Bu_3_P)_2_ (0.16)	—	KOAc (4)	DMA	120 °C	24	63	37	0
9	*anti*-6	Pd(*t*-Bu_3_P)_2_ (0.45)	—	KOAc (12)	DMA	120 °C	24	19	28	0

a Reaction of 6 (0.06 mmol) was performed using a palladium reagent, ligand, and base in a solvent under an argon atmosphere.

b Estimated yield calculated by ignoring the small amount of inseparable impurities in the product.

**Fig. 2:**
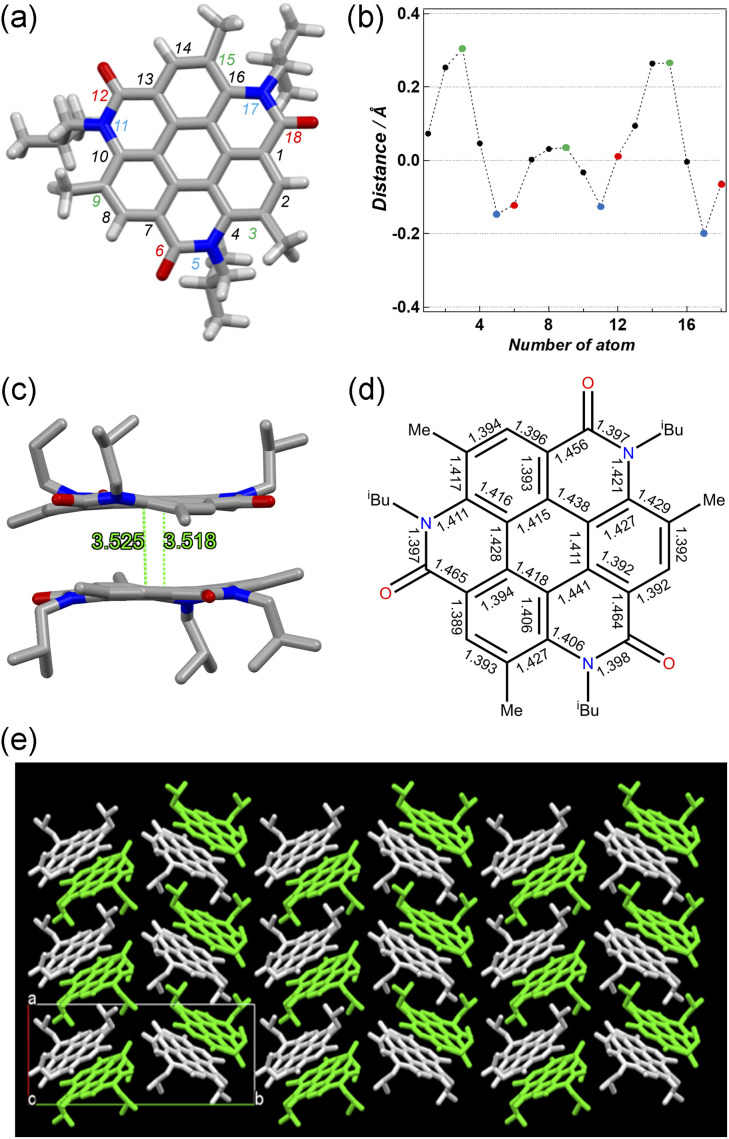
The crystal structure of 3; (a) top view and the numbering of peripheral atoms (nitrogen and oxygen atoms are indicated in blue and red, respectively), (b) the distance between the selected atom and 2D plane based on the central ring, (c) the dimerized molecules with the perpendicular distance (Å) from the center of the central benzene ring in one molecule to the plane of the central benzene ring in the other molecule, and (d) the bond lengths. (e) Packing structure of molecules of 3 viewed down the *c*-axis.

Pale brown plate-like crystals of 3 suitable for X-ray diffraction measurements were obtained by the slow evaporation of a solution of 3 in CH_2_Cl_2_/hexane at room temperature. Fig. 2a shows the crystal structure of 3. The unit-cell dimensions of 3 were *a* = 11.26590(10), *b* = 25.57580(10), *c* = 11.44520(10) Å, and *β* = 116.5650(10)° (Table S1, ESI†).^27^ Four molecules were present in the unit cell (*Z* = 4), and solvent molecules were absent in the crystal lattice. Compound 3 crystallized as a kryptoracemate in the Sohncke space group *P*2_1_ with two enantiomeric molecules in the asymmetric unit (*Z*′= 2).^28,29^ Compared to the core of 3 with those of other coronene analogues,^14–17,19,20^ it exhibited low planarity with a maximum distance of 0.304 Å between the peripheral atom of the core and 2D plane based on the central ring (Fig. 2b). The non-planar structure might be related to the steric repulsion between the *N*-isobutyl group of the amide bond and methyl group of the adjacent benzene ring. In its structure, just two molecules were stacked separately by π–π stacking (*d* = 3.518–3.525 Å, Fig. 2c) and multiple CH⋯O bonds (*d*_C–O_ = 3.177–3.553 Å) (Fig. S1, ESI†). Each of the three isobutyl groups was directed in the opposite direction, and only two molecules in 3 exhibited π–π stacking, just not as some other coronene derivatives with continuous π–π interaction in a one-dimensional columnar structure (Fig. 2e and S2, ESI†).^14–16,20^ Given the structure of 3, alternations of the C–C bond lengths in the central hexagon (1.411–1.441 Å) represented lower aromaticity than that of the benzene ring (Fig. 2d).

**Fig. 3:**
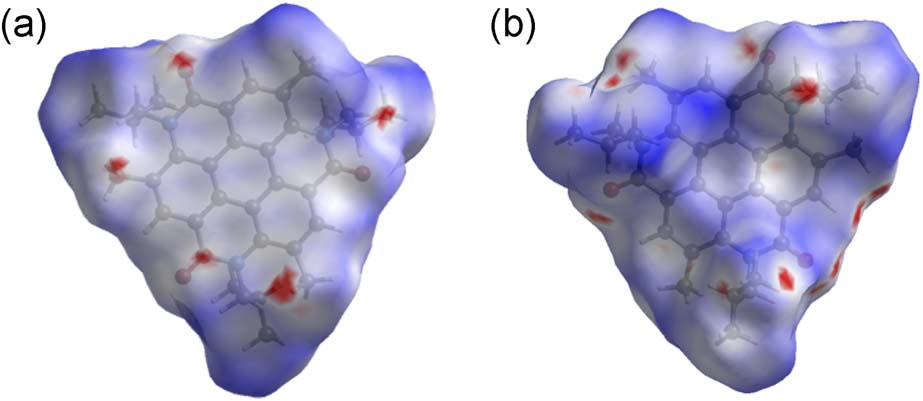
Hirshfeld surface of 3 mapped with *d*_norm_ viewed from (a) the core side and (b) isobutyl group side (mapped over a fixed color scale of −0.222 (red) to 1.823 (blue) a.u.).

Hirshfeld surfaces and energy framework analyses were applied to visually and quantitatively evaluate the intermolecular interactions of 3 in the solid state. CrystalExplorer 17.5 was applied to generate Hirshfeld surfaces and energy framework using the cif file obtained from single crystal X-ray diffraction data of 3.^30,31^ The analysis was visualized by the normalized contact distance (*d*_norm_), which was obtained using a high surface resolution with a static color scale of −0.222 Å (red) to 1.823 Å (blue). The *d*_norm_ is a symmetric function of distances to the surface between nuclei inside and outside the Hirshfeld surface (*d*_i_ and *d*_e_, respectively),^30,31^ relative to their respective van der Waals (vdW) radii. The red areas show the intermolecular contacts less than their vdW radius of the atom, while the blue areas show intermolecular contacts longer than their vdW radii. White areas are the same of their vdW radii. As shown in Fig. 3a, the red areas on the Hirshfeld surface of 3 represent a close intermolecular CH⋯O distance between two coronene molecules. Because the white color represents the distance identical to vdW radii, the white area at the core unit of 3 indicates that the π–π contacts appear over the entire plane of the molecule. The molecular surface corresponding to the isobutyl groups, on the other hand, is represented by blue, indicative of a relatively far distance between them (Fig. 3b). Energy framework analysis supporting these results provides quantitative visualization of intermolecular interactions (Fig. S3, ESI),† which includes the mapping of an interaction between two molecules in the crystal based on the strength of interaction energies (IE). Energy frameworks were constructed from pairwise intermolecular interaction energy calculations using the HF/3-21G in CrystalExplorer 17.5.^30,31^ The calculated total interaction energy includes electrostatic, polarization, dispersion, and exchange-repulsion terms. This is represented as a cylindrical tube, the thickness of which revealed the intensity of the interactions between two molecules (Fig. S4, ESI†). It is most energetically stabilized as IE = −149.3 kJ mol^−1^ when the two molecules interacted closely based on the π–π and CH⋯O interactions (Fig. S3, ESI).† In particular, the dispersion energy provides a strong contribution to its stabilization (IE = −200.4 kJ mol^−1^). The total energy with the other molecules, on the other hand, is considerably weaker and greater than −66.5 kJ mol^−1^.

**Fig. 4:**
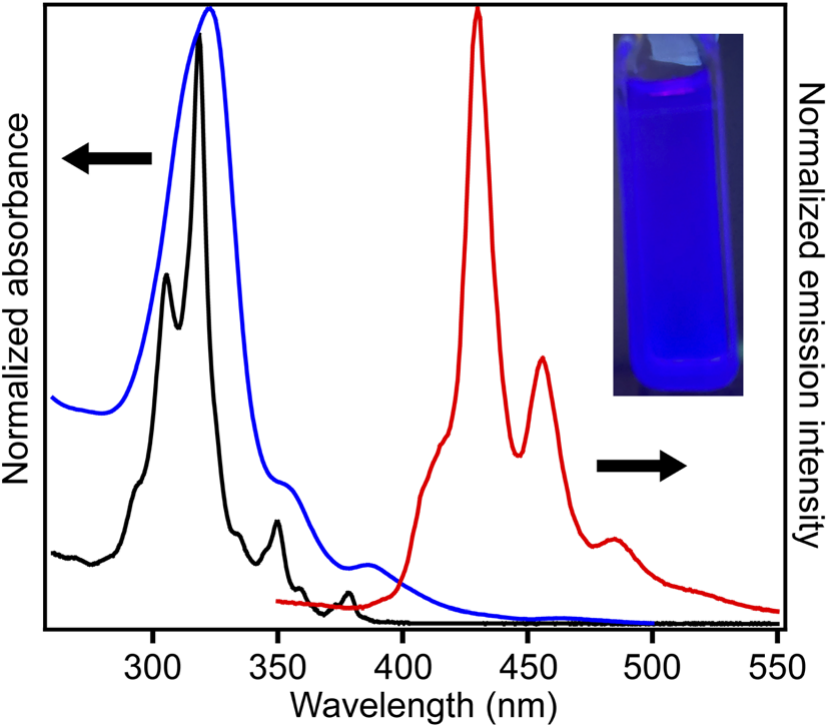
UV-vis absorption (blue line) and fluorescence spectra (red line, excitation at 323 nm) of 3 in CHCl_3_ (5.0 × 10^−4^ M), and UV-vis absorption spectrum of 2 (black line) in CHCl_3_ (1.3 × 10^−5^ M). Inset: photograph of purple luminescence of the solution of 3 in CHCl_3_ (5.0 × 10^−4^ M) under a 365 nm UV lamp.

**Fig. 5:**
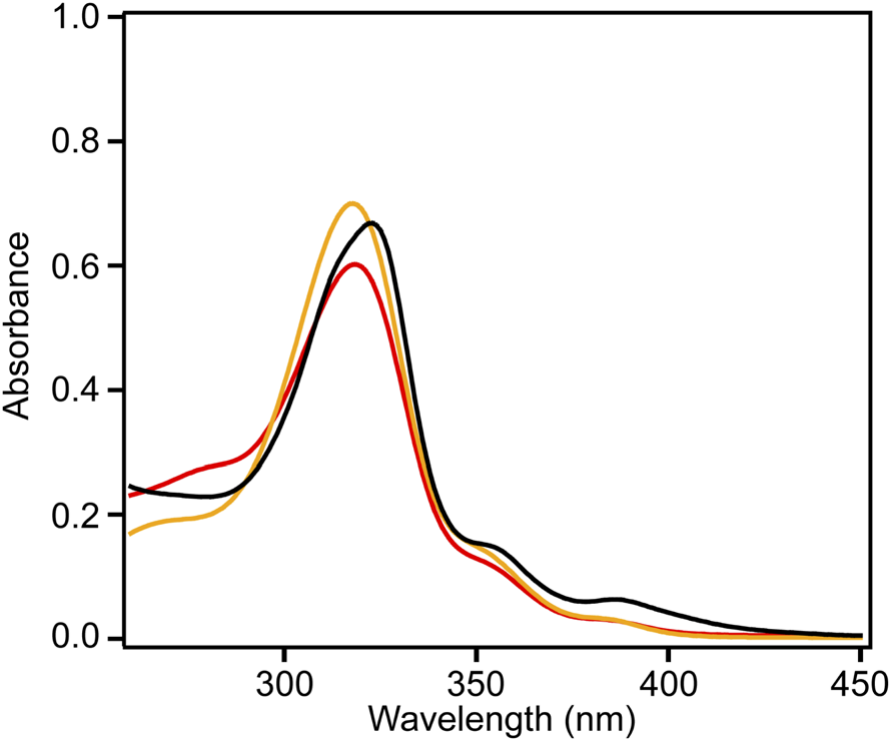
Absorption spectra of 3 in chloroform (black), tetrahydrofuran (red), and ethyl acetate (orange) (5.0 × 10^−4^ M).

Fig. 4 shows the UV-vis absorption spectra of 2 and 3 and the emission spectrum of 3 in CHCl_3_ solutions. Compound 3 exhibited an absorption band at 324 nm, and a low-intensity shoulder peak at ∼390 nm in the lowest-energy region. Luminescence with emission maxima at 429, 455, and 484 nm was observed after excitation at 323 nm, corresponding to a Stokes shift of *ca.* 100 nm. The absorption spectrum of 2 without methyl substituents exhibited maxima at 319 and 305 nm and weak maxima at 350 and 379 nm.^21^ The absorption spectrum of 3 was similar to that of 2, indicating the similar electronic structure of these compounds. The UV-vis absorption spectra of 3 in tetrahydrofuran and ethyl acetate were similar to that in chloroform, and a notable solvent effect was not observed (Fig. 5). In the cyclic voltammetry (CV) shown in Fig. S5 (ESI†), 3 did not exhibit any oxidation or reduction peak in the range of −2.1 V to +1.0 V *vs.* Fe/Fe^+^, indicating that 3 has a higher energy level LUMO than the reported amide bond-containing coronene 1.^17^ This was probably due to the reduction of the π-conjugation induced by the low planarity of 3 or by the lack of four benzene units linked to amide nitrogen in 3 compared to 1.

## Conclusions

The coronene amide analogue 3 was efficiently synthesized and its properties were discussed. Tetrasubstituted aromatic compound 9 was prepared by the regioselective bromination of 8. Subsequent four-step transformations according to our previous study afforded *syn*-6 and *anti*-6. The palladium-mediated intramolecular biarylation of 6 proceeded with Pd(*t*-Bu_3_P)_2_ and KOAc in DMA to afford coronene amide analogue 3 in good yield. Single-crystal X-ray analysis revealed that the core structure of 3 is not planar, possibly derived from steric repulsions between isobutyl and methyl groups. The UV-vis absorption spectra indicated that 2 and 3 had the similar electronic structure. The sequential cyclic amide formation/intramolecular biarylation procedure could be applied to the synthesis of other amide bond-containing polycyclic aromatic compounds. Currently, the synthesis of various polycyclic aromatic compounds using this method and studies on their structure and properties are underway.

## Experimental section

### General experimental methods

Column chromatography was performed on silica gel (neutral silica gel 60N, 63–210 μm, Kanto Chemical) with a specified solvent. ^1^H and ^13^C NMR spectra were obtained on a JEOL ECA-500 instrument. The internal standards of the ^1^H and ^13^C NMR spectra were tetramethylsilane (0.00 ppm) and the midpoint of CDCl_3_ (77.0 ppm) in CDCl_3_, respectively. IR spectra were recorded on a JASCO FT/IR-470 plus. Electrospray ionization (ESI) mass spectra were recorded on a Thermo Fisher Scientific Q Exactive Hybrid Quadrupole-Orbitrap Mass Spectrometer. UV-vis spectra were recorded on a JASCO V-630 spectrophotometer. Luminescence spectra were recorded on a SHIMADZU RF-5300PC spectrofluorophotometer. Cyclic voltammetry was performed on an ALS Model 600C electrochemical analyzer. X-ray data were collected on a Rigaku XtaLAB P200 diffractometer with multi-layer mirror monochromated Cu Kα radiation and a hybrid photon counting detector (PILATUS 200K) at −180 °C. The data were corrected for Lorentz and polarization effects. An empirical absorption correction based on the multiple measurement of equivalent reflections was applied using CrystalClear program. The structures were solved by direct method (SHELXT^32^) and refined by full-matrix least squares fitting on *F*^2^ (SHELXL Version 2018/3 (ref. 33,34)) using all data. All non-hydrogen atoms were refined anisotropically. The aromatic protons of 3 were located from difference syntheses and ride on the parent atoms. The other hydrogen atoms were located on the calculated positions. All calculations were performed using CrystalStructure crystallographic software package except for refinement, which was performed using SHELXL. The results are shown in Table S1 (ESI).† The absolute configuration could not be decided from the Flack parameters.^35,36^ Crystallographic data have been deposited with the Cambridge Crystallographic Data Center as supplementary publication number CCDC 2130884. These data can be obtained free of charge from the Cambridge Crystallographic Data Center *via*www.ccdc.cam.ac.uk/structures/.

#### Synthesis of 9

To commercially available methyl 3-methyl-4-nitrobenzoate (8, 3.00 g, 15.4 mmol) was added NBS (3.28 g, 18.4 mmol), Pd(OAc)_2_ (449 mg, 2.00 mmol), and 1-fluoropyridinium triflate (4.56 g, 18.4 mmol) under an argon atmosphere. The flask was degassed under reduced pressure and filled with argon three times. Then dry (CH_2_Cl)_2_ (30 mL) and TfOH (10.7 mL, 121 mmol) were added, and the mixture was stirred at 60 °C for 6 h under an argon atmosphere and quenched with saturated aqueous NaHCO_3_ at room temperature. The aqueous layer was extract three times with CH_2_Cl_2_. The combined organic layer was washed with brine, dried over anhydrous Na_2_SO_4_, and concentrated under reduce pressure. Purification with silica gel column chromatography (AcOEt/hexane = 1/24) gave 9 (3.48 g, 82%) as a white solid. ^1^H NMR (500 MHz, CDCl_3_) *δ* 8.23 (s, 1H), 7.76 (s, 1H), 3.97 (s, 3H), 2.58 (s, 3H); ^13^C NMR (126 MHz, CDCl_3_) *δ* 165.1, 150.3, 136.0, 135.2, 132.5, 129.9, 118.9, 53.0, 19.8; HRMS (ESI^−^) calculated for [C_9_H_7_BrNO_4_^−^] 271.9563 [M − H]^−^, found 271.9570; IR (KBr) 3029, 1724, 1567, 1524, 1429, 1371, 1264, 1205, 1135, 985, 914, 838, 795, 756, 585, 495 cm^−1^; mp 103.4–105.6 °C.

#### Synthesis of 10

SnCl_2_·2H_2_O (1.27 g, 5.46 mmol) was added to a solution of 9 (299 mg, 1.09 mmol) in EtOH (1.5 mL). The reaction mixture was stirred at 70 °C for 20 min. After addition of saturated aqueous NaHCO_3_ and filtration with Celite pad with AcOEt, the filtrate was extracted three times with AcOEt. The combined organic layer was washed with brine, dried over anhydrous Na_2_SO_4_, and concentrated under reduce pressure. Purification with silica gel column chromatography (AcOEt/hexane = 1/24) afforded 10 (267 mg, quantitative yield) as a white solid. ^1^H NMR (500 MHz, CDCl_3_) *δ* 7.67 (s, 1H), 6.91 (s, 1H), 3.99 (br s, 2H), 3.86 (s, 3H), 2.12 (s, 3H); ^13^C NMR (126 MHz, CDCl_3_) *δ* 166.0, 148.9, 134.2, 121.1, 120.3, 119.4, 119.3, 51.8, 16.6; HRMS (ESI^+^) calculated for [C_9_H_11_BrNO_2_^+^] 243.9968 [M + H]^+^, found 243.9963; IR (KBr) 3488, 3368, 1697, 1628, 1560, 1503, 1434, 1333, 1259, 1185, 1118, 992, 930, 768 cm^−1^; mp 128.1–129.5 °C.

#### Synthesis of 11

To a solution of 10 (137 mg, 0.561 mmol) and isobutyraldehyde (42 mg, 0.58 mmol) in dry (CH_2_Cl)_2_ (2.0 mL), NaBH(OAc)_3_ (131 mg, 0.618 mmol) was added at room temperature under a nitrogen atmosphere. After the reaction mixture was stirred for 3 h at room temperature, additional isobutyraldehyde (43 mg, 0.59 mmol) and NaBH(OAc)_3_ (130 mg, 0.613 mmol) were added. The mixture was stirred for 1 h at room temperature and quenched with saturated aqueous NaHCO_3_. The aqueous layer was extracted three times with AcOEt, and the combined organic layer was washed with brine and dried over anhydrous Na_2_SO_4_. Purification with silica gel column chromatography (AcOEt/hexane = 1/10) gave 11 (167 mg, 99%) as a white solid. ^1^H NMR (500 MHz, CDCl_3_) *δ* 7.67 (d, *J* = 0.6 Hz, 1H), 6.78 (s, 1H), 3.95 (br s, 1H), 3.86 (s, 3H), 3.00 (dd, *J* = 5.4 and 6.9 Hz, 2H), 2.09 (s, 3H), 1.94 (nonet, *J* = 6.7 Hz, 1H), 1.01 (d, *J* = 6.6 Hz, 6H); ^13^C NMR (126 MHz, CDCl_3_) *δ* 166.1, 149.9, 133.5, 122.3, 119.7, 117.0, 114.5, 51.7, 51.0, 27.9, 20.4, 16.7; HRMS (ESI^+^) calculated for [C_13_H_19_BrNO_2_^+^] 300.0594 [M + H]^+^, found 300.0590; IR (KBr) 3420, 2152, 1601, 1561, 1518, 1436, 1393, 1339, 1265, 1177, 1124, 930, 836, 770, 652, 500 cm^−1^; mp 73.6–75.7 °C.

#### Synthesis of 12

To a solution of 11 (1.32 g, 4.41 mmol) in MeOH/H_2_O (1/1, 25 mL), KOH (977 mg, 17.4 mg) was added at room temperature. The reaction mixture was stirred at 75 °C for 2 h, then 1 M hydrochloric acid was added at room temperature until pH became 1. The aqueous layer was extracted three times with AcOEt. The combined organic layer was washed with brine, dried over anhydrous MgSO_4_, and concentrated under reduce pressure. Purification with silica gel column chromatography (AcOEt/hexane = 1/2) afforded 12 (1.24 g, 98%) as a white solid. ^1^H NMR (500 MHz, CDCl_3_) *δ* 7.82 (d, *J* = 0.6 Hz, 1H), 6.81 (s, 1H), 3.03 (d, *J* = 6.9 Hz, 2H), 2.10 (s, 3H), 1.95 (nonet, *J* = 6.7 Hz, 1H), 1.02 (d, *J* = 6.6 Hz, 6H); ^13^C NMR (126 MHz, CDCl_3_) *δ* 170.7, 150.6, 134.6, 123.3, 119.8, 115.6, 114.7, 51.0, 27.9, 20.4, 16.7; HRMS (ESI^−^) calculated for [C_12_H_15_BrNO_2_^−^] 284.0291 [M − H]^−^, found 284.0298; IR (KBr) 3436, 2927, 1671, 1599, 1559, 1473, 1346, 1282, 1255, 1180, 960, 835, 759, 650 cm^−1^; mp 183.6–185.7 °C.

#### Synthesis of 6

To a solution of 12 (1.23 g, 4.31 mmol) in pyridine (25.0 mL), SiCl_4_ (0.70 mL, 6.1 mmol) was slowly added at 0 °C under an argon atmosphere. The reaction mixture was stirred at 120 °C for 42 h, cooled to room temperature, and concentrated under reduce pressure. After addition of CH_2_Cl_2_ to the residue, the mixture was washed with 1 M hydrochloric acid, dried over anhydrous MgSO_4_, and concentrated under reduced pressure. Purification with silica gel column chromatography (AcOEt/hexane gradient = 2/5, 1/1, 3/1) gave *syn*-6 (591 mg, 51%) as a white solid and *anti*-6 (241 mg, 21%) as a white solid.

*syn*-6: ^1^H NMR (500 MHz, CDCl_3_) *δ* 7.64 (s, 3H), 6.85 (s, 3H), 4.18 (dd, *J* = 9.7 and 13.2 Hz, 3H), 2.77 (dd, *J* = 4.9 and 13.5 Hz, 3H), 2.12 (s, 9H), 1.87–1.79 (m, 3H), 1.14 (d, *J* = 6.6 Hz, 9H), 0.93 (d, *J* = 6.6 Hz, 9H); ^13^C NMR (126 MHz, CDCl_3_) *δ* 167.9, 141.1, 138.8, 134.3, 132.9, 127.8, 115.9, 54.7, 26.9, 20.6, 20.4, 17.1; HRMS (ESI^+^) calculated for [C_36_H_42_^79^Br^81^Br_2_N_3_O_3_Na^+^] 826.0649 [M + Na]^+^, found 826.0652; IR (KBr) 3854, 3735, 3469, 2958, 1657, 1599, 1558, 1487, 1402, 1336, 1297, 1162, 1126, 892 cm^−1^; mp 313.0–314.8 °C.

*anti*-6: ^1^H NMR (500 MHz, CDCl_3_) *δ* 7.67 (s, 1H), 7.19 (s, 1H), 7.16 (s, 1H), 6.98 (s, 1H), 6.89 (s, 1H), 6.68 (s, 1H), 4.39 (dd, *J* = 9.5 and 13.2 Hz, 1H), 4.05 (dd, *J* = 8.7 and 13.3 Hz, 1H), 3.68 (dd, *J* = 7.9 and 13.3 Hz, 1H), 3.45 (dd, *J* = 6.9 and 13.5 Hz, 1H), 3.08 (dd, *J* = 6.0 and 13.2 Hz, 1H), 2.79 (dd, *J* = 5.3 and 13.3 Hz, 1H), 2.36 (s, 3H), 2.26 (s, 3H), 1.98 (s, 3H), 1.95–1.79 (m, 3H), 1.07–1.04 (m, 6H), 1.02 (d, *J* = 6.6 Hz, 3H), 1.00–0.98 (m, 6H), 0.93 (d, *J* = 6.9 Hz, 3H); ^13^C NMR (126 MHz, CDCl_3_) *δ* 168.2, 168.0, 167.8, 141.4, 140.9, 140.5, 138.3, 137.0, 136.13, 136.11, 135.7, 135.6, 135.2, 134.3, 133.5, 131.9, 131.6, 131.0, 115.7, 114.7, 114.1, 56.3, 55.4, 54.3, 27.3, 27.1, 26.9, 20.62, 20.57, 20.5, 20.4, 20.3, 20.2, 18.6, 17.9, 17.6; HRMS (ESI^+^) calculated for [C_36_H_42_^79^Br^81^Br_2_N_3_O_3_Na^+^] 826.0649 [M + Na]^+^, found 826.0657; IR (KBr) 3735, 2959, 1655, 1597, 1558, 1487, 1398, 1332, 1295, 1164, 1125, 1035, 893, 736, 643 cm^−1^; mp 191.6–193.9 °C.

#### General procedure of Pd-mediated biarylation (Table 1, entry 8)

To a mixture of *syn*-6 (50.0 mg, 0.0622 mmol), Pd(*t*-Bu_3_P)_2_ (5.0 mg, 0.0098 mmol), and KOAc (25.6 mg, 0.261 mmol), dry DMA (1.8 mL) was added under an argon atmosphere. The flask was degassed under reduced pressure and filled with argon three times and then stirred at 120 °C for 24 h. After cooling to room temperature and filtering with Celite pad with AcOEt, the filtrate was wash with H_2_O, 1 M hydrochloric acid, and brine. The organic layer was dried over anhydrous MgSO_4_ and concentrated under reduced pressure. Purification with silica gel column chromatography (AcOEt/toluene = 1/9) gave 3 (22.1 mg, 63%) as a pale brown solid and 13 (13.2 mg, 37%) as an orange solid.

Compound 3: ^1^H NMR (500 MHz, CDCl_3_) *δ* 8.63 (s, 3H), 4.77 (br s, 6H), 2.99 (s, 9H), 1.87 (m, 3H), 0.69 (m, 18H); ^13^C NMR (126 MHz, CDCl_3_) *δ* 163.6, 139.7, 133.4, 125.5, 124.0, 117.2, 112.6, 51.9, 27.7, 24.9, 19.4; HRMS (ESI^+^) calculated for [C_36_H_39_N_3_O_3_Na^+^] 584.2884 [M + Na]^+^, found 584.2887; IR (KBr) 3854, 3839, 3735, 3676, 3649, 3567, 1716, 1698, 1647, 1617, 1558, 1541, 1508, 1457, 419 cm^−1^; mp 358.2–360.4 °C.

Compound 13: ^1^H NMR (500 MHz, CDCl_3_) *δ* 8.44 (d, *J* = 0.6 Hz, 1H), 7.79 (d, *J* = 0.6 Hz, 1H), 6.17 (d, *J* = 8.9 Hz, 1H), 5.99 (dd, *J* = 1.4 and 8.8 Hz, 1H), 5.71 (t, *J* = 1.4 Hz, 1H), 4.32–4.28 (m, 1H), 3.98–3.87 (m, 3H), 3.75 (dd, *J* = 9.6 and 13.9 Hz, 1H), 2.92 (dd, *J* = 6.2 and 13.9 Hz, 1H), 2.69 (s, 3H), 2.53 (s, 3H), 2.30–2.23 (m, 1H), 2.14–2.04 (m, 1H), 1.84–1.79 (m, 1H), 1.48 (d, *J* = 1.4 Hz, 3H), 1.07–1.00 (m, 12H), 0.88 (t, *J* = 7.0 Hz, 3H); ^13^C NMR (126 MHz, CDCl_3_) *δ* 178.6, 168.6, 163.9, 146.1, 144.9, 144.3, 136.3, 134.8, 132.6, 130.2, 129.1, 127.2, 126.7, 126.6, 124.9, 123.4, 121.2, 120.9, 117.7, 68.4, 57.5, 52.0, 49.2, 48.6, 29.4, 28.4, 26.6, 22.9, 20.8, 20.3, 19.71, 19.69, 19.62, 15.4; HRMS (ESI^+^) calculated for [C_36_H_41_N_3_O_3_Na^+^] 586.3041 [M + Na]^+^, found 586.3047; IR (KBr) 3735, 3446, 2959, 1702, 1653, 1547, 1457, 1383, 1156, 1047, 913, 806, 748, 680, 575, 417 cm^−1^; mp 124.2–126.5 °C.

## Conflicts of interest

There are no conflicts to declare.

## Supplementary Material

RA-012-D2RA04035B-s001

RA-012-D2RA04035B-s002
