# Supplementary material for: Synthesis, crystal structure, and properties of methyl-substituted coronene amide analogue
Source: RSC Adv. 2022 Sep 16;12(40):26411–7. doi: 10.1039/d2ra04035b (PMC9479769; doi:10.1039/d2ra04035b)

Electronic Supplementary Material (ESI) for RSC Advances.
This journal is © The Royal Society of Chemistry 2022

Supplementary Information

Synthesis, crystal structure, and properties of methyl-substituted coronene amide analogue

Kenta Rakumitsu,[a] Miho Fujii,[a] Sotaro Kusumoto,[b] Shoko Kikkawa,[c] Isao Azumaya,[c] Akihiro Yokoyama*[a]

[a] *Faculty of Science and Technology, Seikei University, 3-3-1 Kichijoji-kitamachi, Musashino, Tokyo 180-8633, Japan*
[b] *Department of Material and Life Chemistry, Faculty of Engineering, Kanagawa University, 3-27-1 Rokkakubashi, Kanagawa-ku, Yokohama 221-8686, Japan*
[c] *Faculty of Pharmaceutical Sciences, Toho University, 2-2-1 Miyama, Funabashi, Chiba 274-8510, Japan*

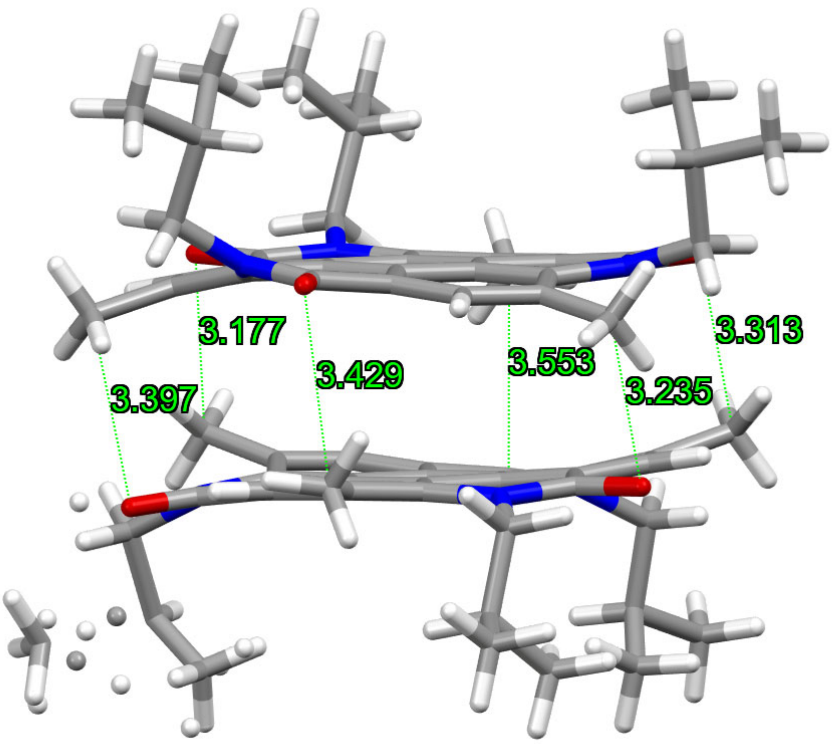

Fig. S1. Multiple CH_3···O contacts (with distance (Å) between C and O) in the stacking molecules.

*Corresponding author.
E-mail address: ayokoyama@st.seikei.ac.jp (A. Yokoyama)

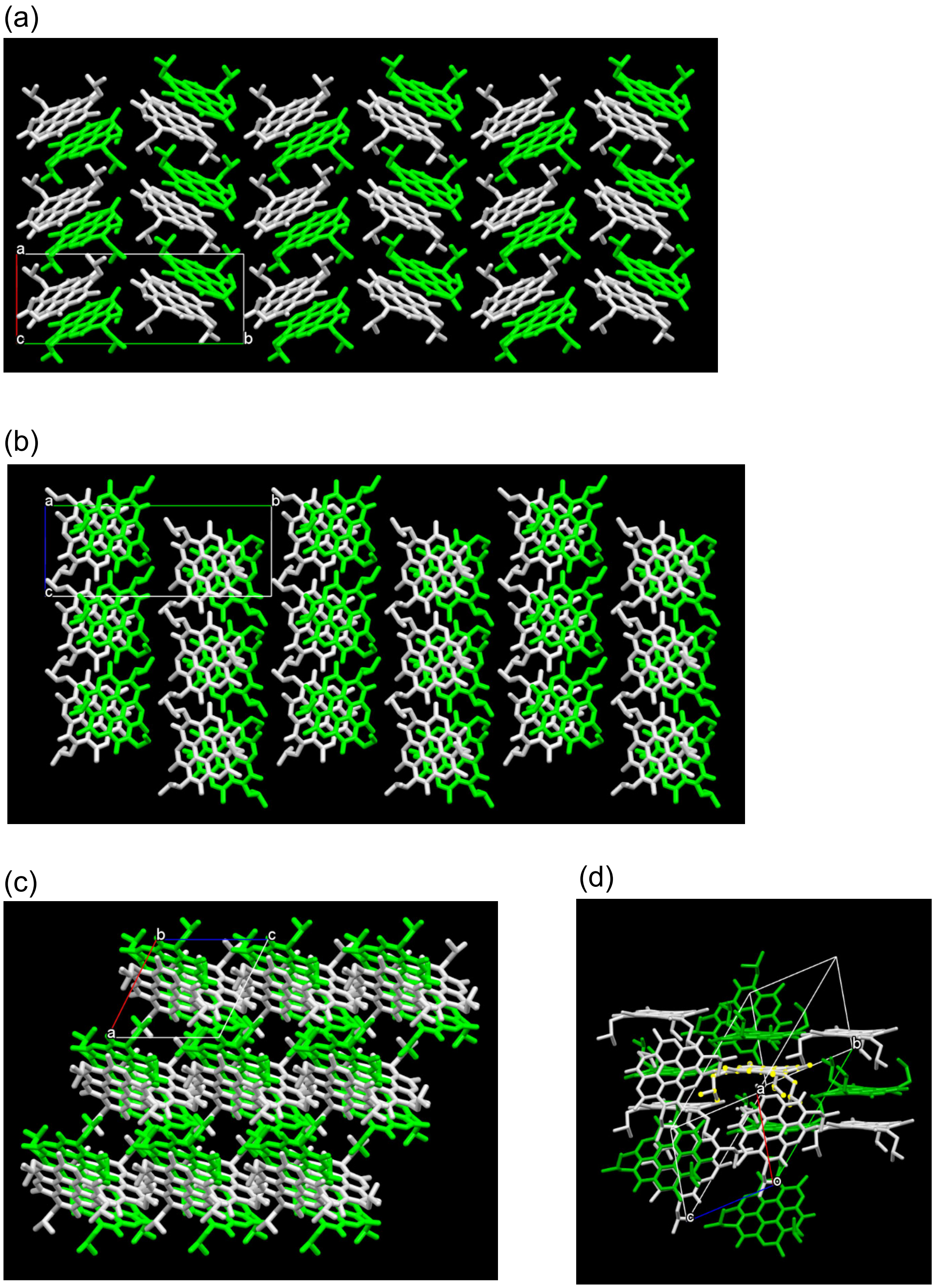

Fig. S2. Packing structure of **3** with symmetry operation viewed down (a) *c*-axis, (b) *b*-axis, and (c) *a*-axis. (d) Packing structure around one of **3** (the same direction as Fig. S3).

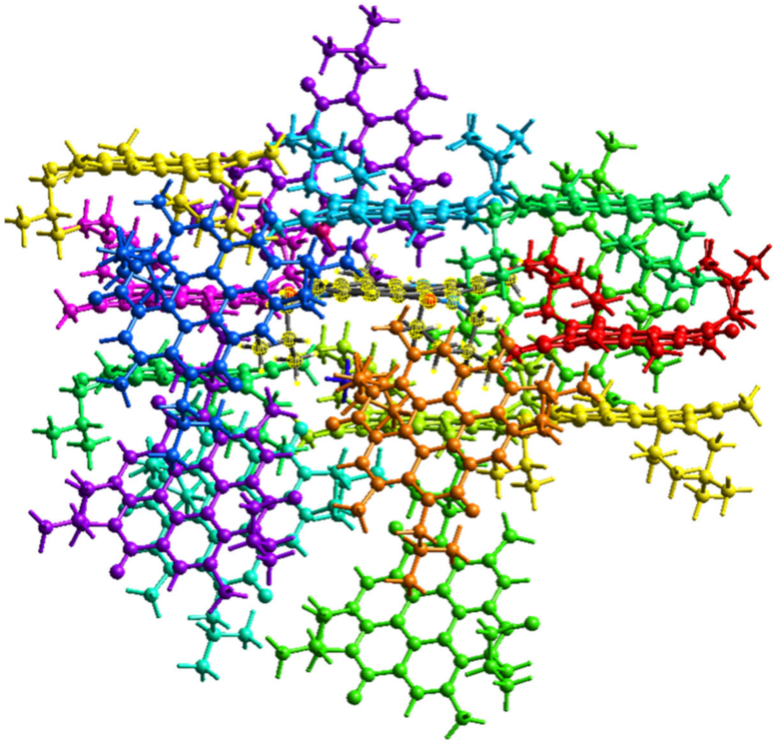

Crystal | Atoms | Surface | Energies

Interaction Energies (kJ/mol)
R is the distance between molecular centroids (mean atomic position) in Å.

Total energies, only reported for two benchmarked energy models, are the sum of the four energy components, scaled appropriately (see the scale factor table below)

	N	Symop	R	Electron Density	E_ele	E_pol	E_dis	E_rep	E_tot
	1	-	10.40	HF/3-21G	-29.0	-15.4	-63.2	36.9	-66.5
	1	-	10.58	HF/3-21G	-10.4	-8.0	-63.3	33.7	-45.5
	2	x, y, z	11.94	HF/3-21G	-0.4	-0.7	-17.1	8.6	-9.3
	1	-	8.29	HF/3-21G	-9.5	-6.0	-90.3	41.5	-61.3
	2	-x, y+1/2, -z	15.42	HF/3-21G	0.1	-0.0	-2.8	0.1	-2.4
	2	x, y, z	11.45	HF/3-21G	-4.4	-2.2	-23.3	10.7	-18.3
	1	-	12.28	HF/3-21G	-4.9	-3.7	-16.5	8.5	-15.3
	1	-	4.93	HF/3-21G	-38.2	-12.0	-200.4	96.2	-149.3
	1	-	12.66	HF/3-21G	0.2	-1.0	-15.1	7.3	-8.2
	1	-	5.62	HF/3-21G	0.0	nan	0.0	0.0	nan
	2	-x, y+1/2, -z	14.37	HF/3-21G	0.0	-0.5	0.0	0.0	-0.3
	1	-	11.47	HF/3-21G	-25.3	-13.6	-34.0	35.4	-36.6
	1	-	9.14	HF/3-21G	0.3	-0.5	-13.8	6.0	-7.6

⟵ stacking pair

Scale factors for benchmarked energy models
See Mackenzie et al. IUCrJ (2017)

Energy Model	k_ele	k_pol	k_disp	k_rep
CE-HF ... HF/3-21G electron densities	1.019	0.651	0.901	0.811
CE-B3LYP ... B3LYP/6-31G(d,p) electron densities	1.057	0.740	0.871	0.618

Fig. S3. The interaction energies of various pairwise molecules estimated using CE-HF/3-21G in CrystalExplorer 17.5.[1] Total interaction energy includes electrostatic, polarization, dispersion, and exchange-repulsion terms. The viewing direction is the same as in Fig S2d.

(a)

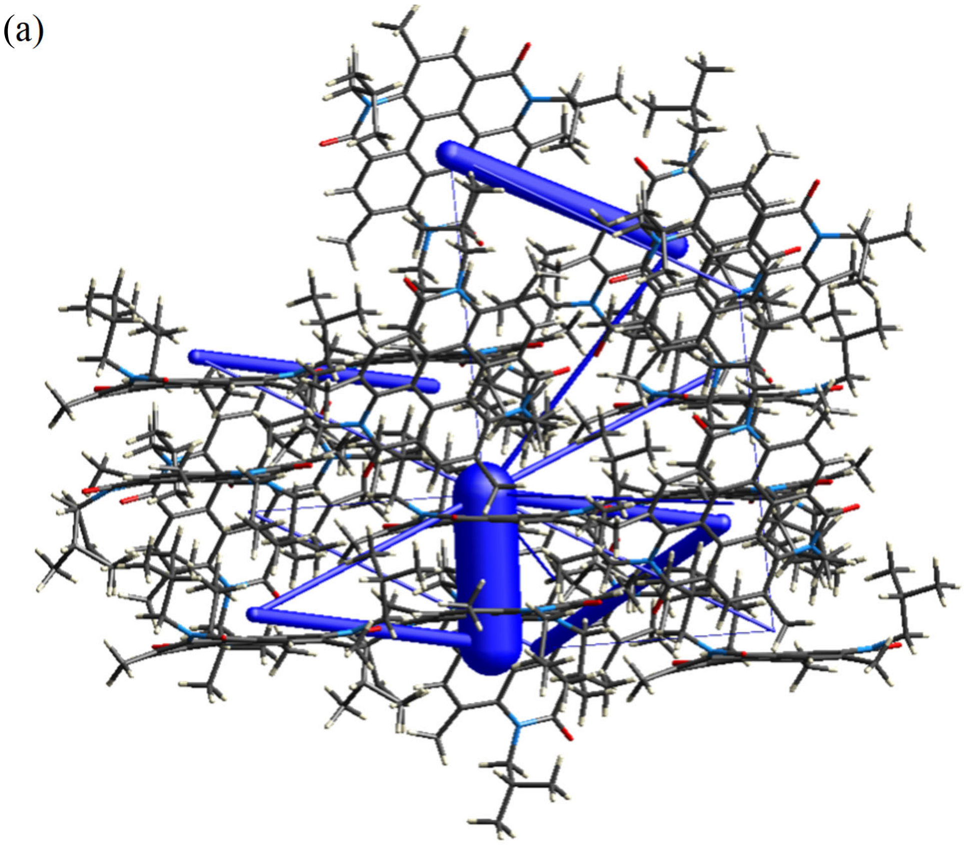

(b)

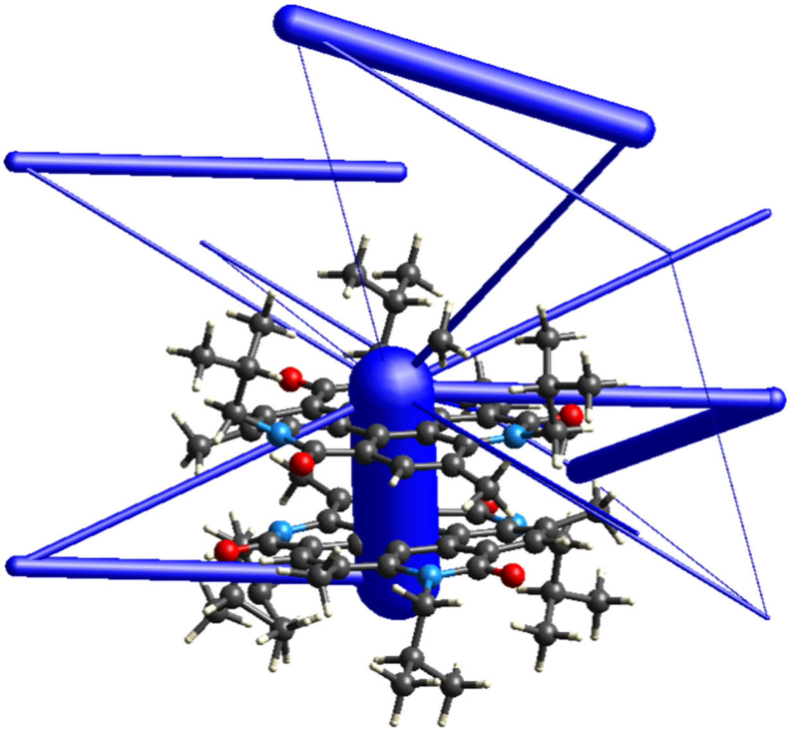

Fig. S4. Energy frameworks of **3** in (a) total interaction strengths and (b) extracted dimers. Energy frameworks were constructed from pairwise intermolecular interaction energy calculations (at crystal geometry).

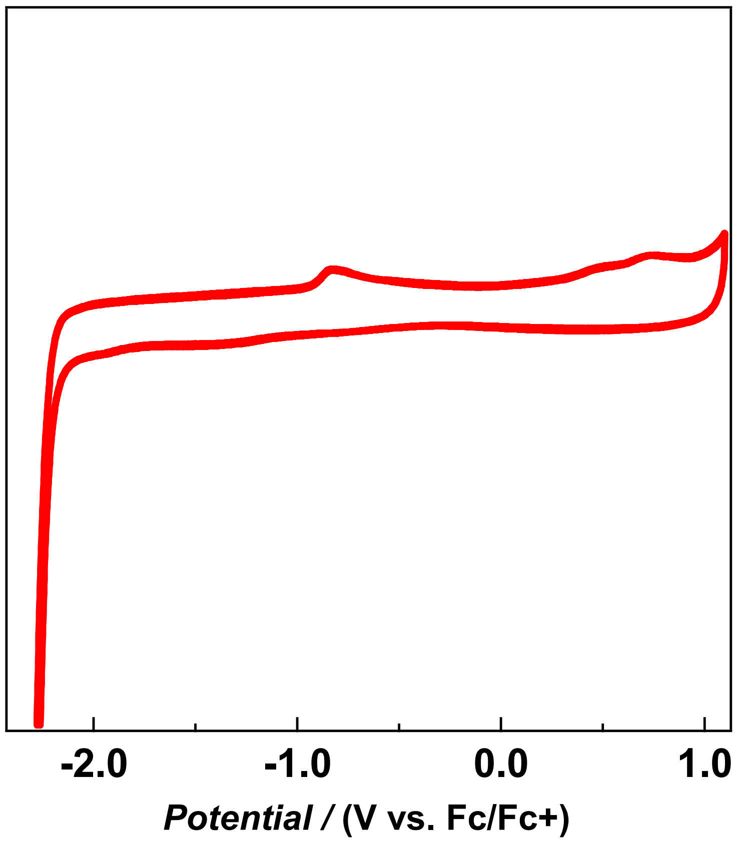

Fig. S5. Cyclic voltammogram of **3** measured in CH_2Cl_2 (0.1 M *n*-Bu_4NPF_6) at a scan rate of 0.1 V/s.

Table S1. Crystallographic data and refinement parameters for **3**.

One of isobutyl groups showed positional disorder. Two possible positions having different orientations were assumed with 50%/50% probabilities for C37A, C39A and C37B, C39B, respectively.

Formula	$C_{36}H_{39}N_3O_3$
M_r	561.72
Crystal system	Monoclinic
Space group	$P2_1$
a / Å	11.26590(10)
b / Å	25.57580(10)
c / Å	11.44520(10)
α / deg	90
β / deg	116.5650(10)
γ / deg	90
V / Å^3	2949.60(5)
Z	4
Z'	2
Temperature / K	93
Goodness-of-fit on F^2	1.041
R_1 [$I > 2\sigma(I)$] on F	0.0383
wR_2 (all data) on F^2	0.1035
Reflection collected (all data)	39543
No. of reflections [$I > 2\sigma(I)$]	10048
R_{int}	0.0225
Abs. corr.	multi-scan
Flack parameter	0.55(3)
Radiation type	Cu$K\alpha$ (λ = 1.54187 Å)
T_{max}	0.838
T_{min}	0.981
$2\theta_{max}$	68.228
μ /mm^{-1}	0.637
CCDC No.	2130884

References

1 Spackman, P. R.; Turner, M. J.; McKinnon, J. J.; Wolff, S. K.; Grimwood, D. J.; Jayatilaka, D.; Spackman, M. A. *J. Appl. Cryst.* **2021**, *3*, 1006–1011.

^{1}H NMR of **9** ($CDCl_3$, 500 MHz)

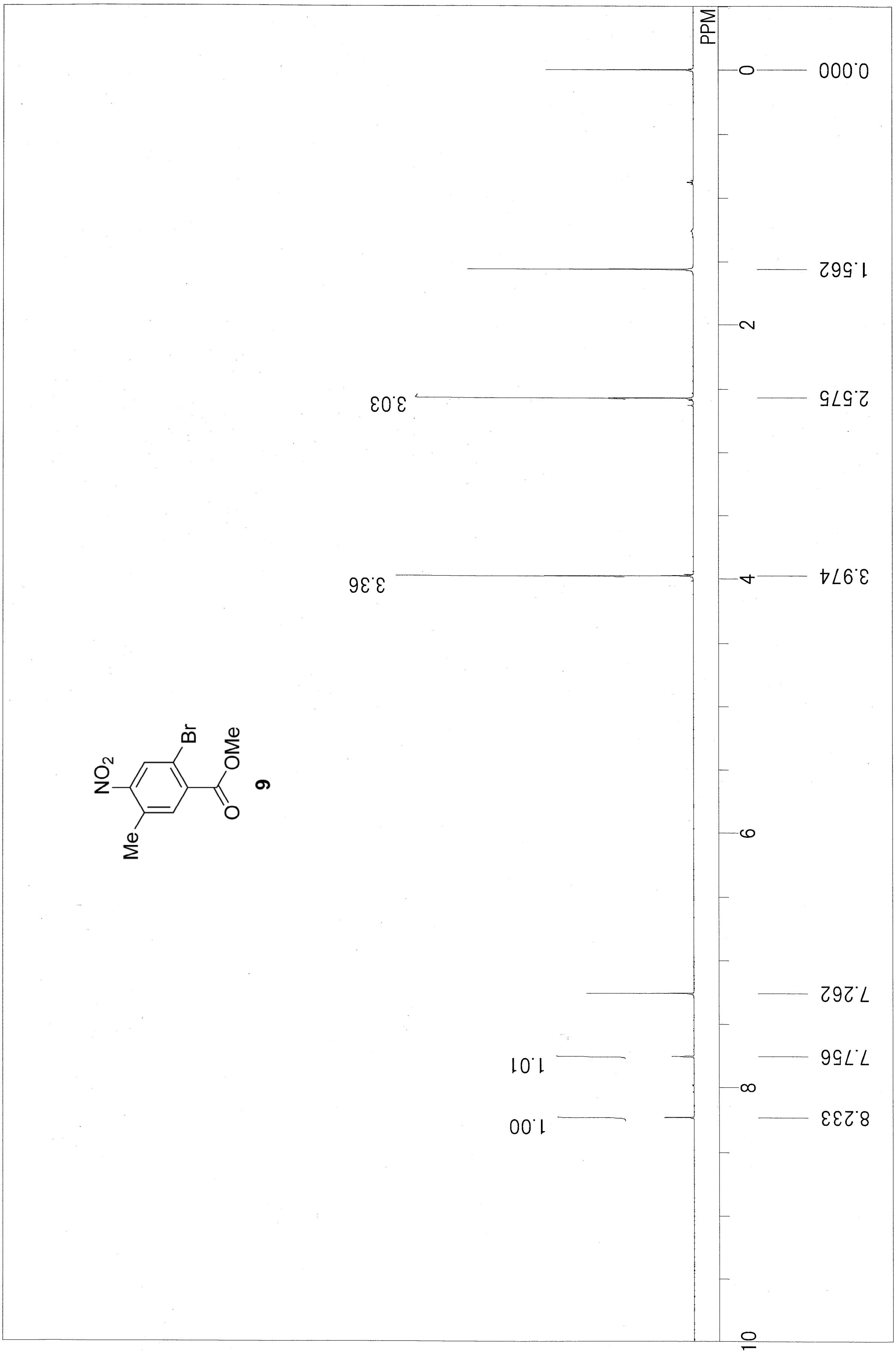

^{13}C NMR of **9** ($CDCl_3$, 126 MHz)

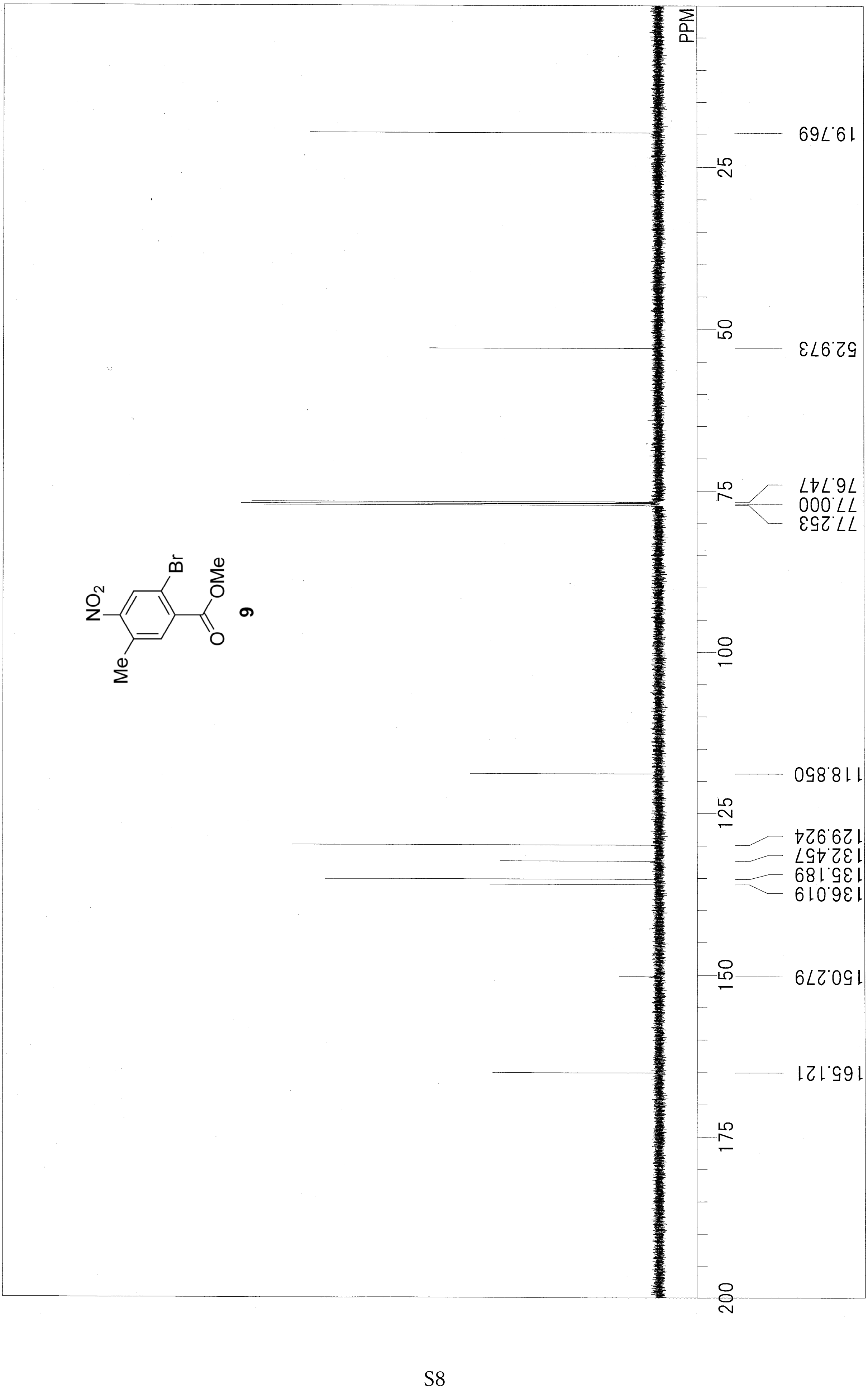

^{1}H NMR of **10** ($CDCl_3$, 500 MHz)

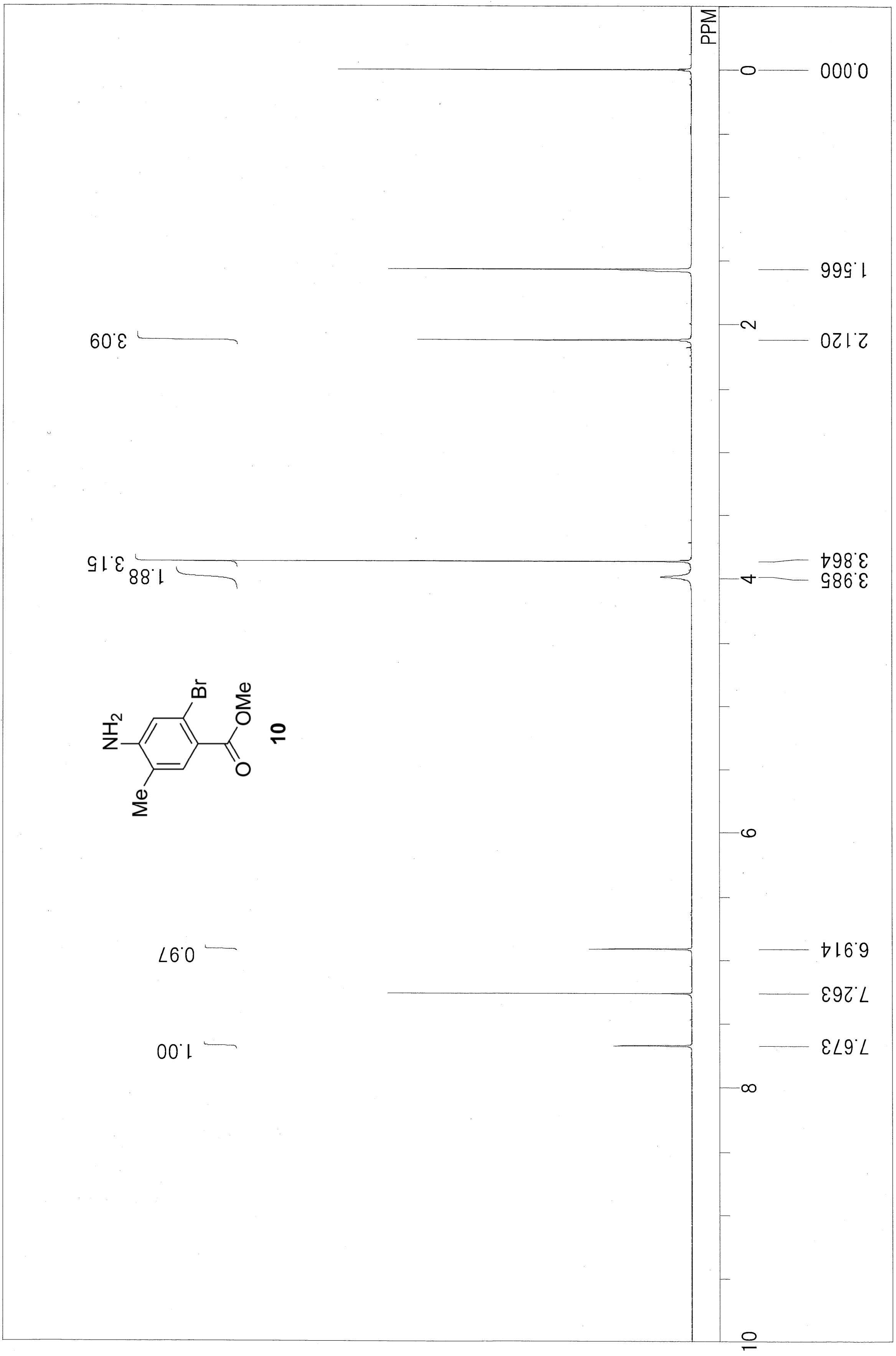

^{13}C NMR of **10** ($CDCl_3$, 126 MHz)

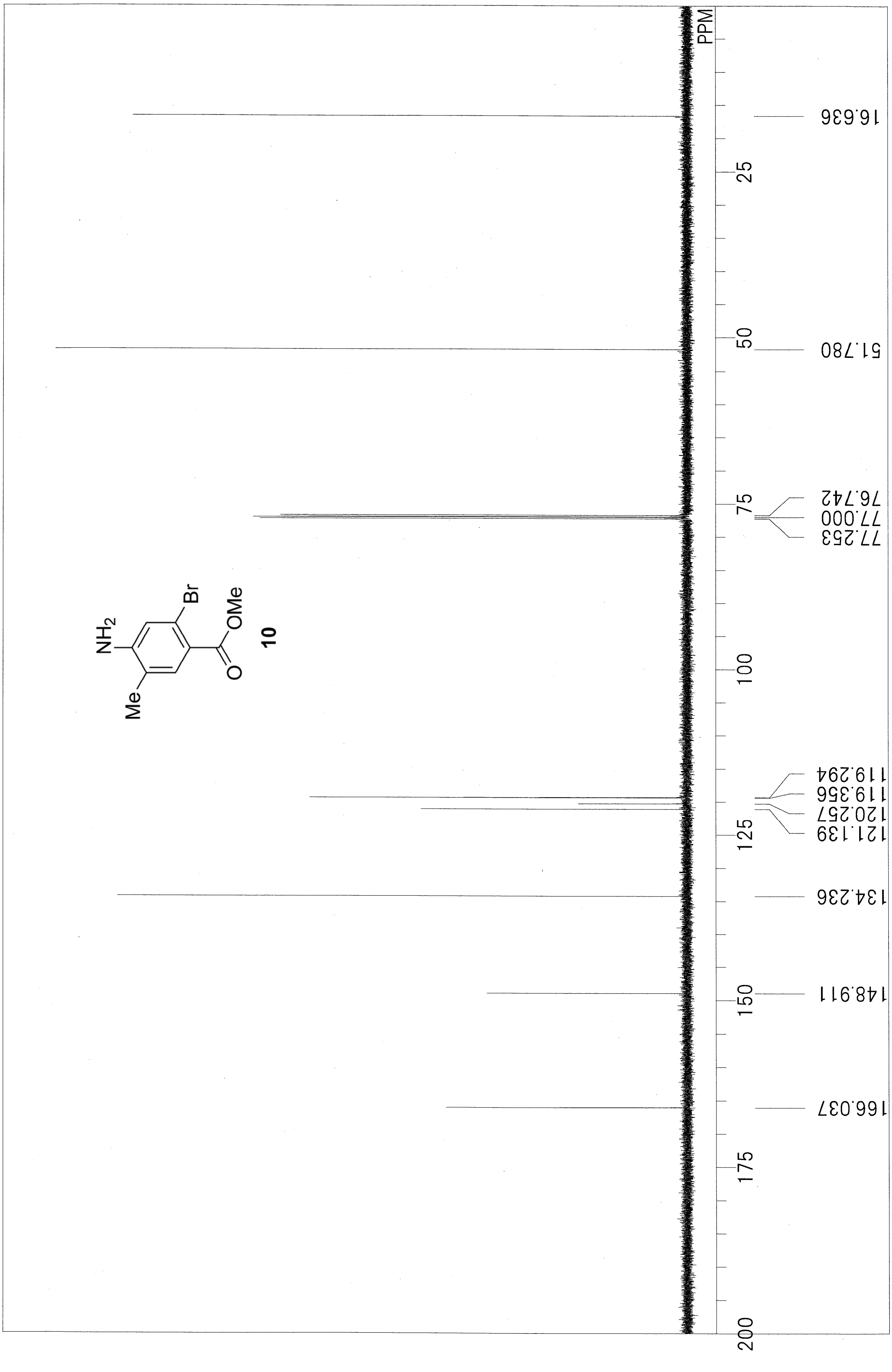

^{1}H NMR of **11** ($CDCl_3$, 500 MHz)

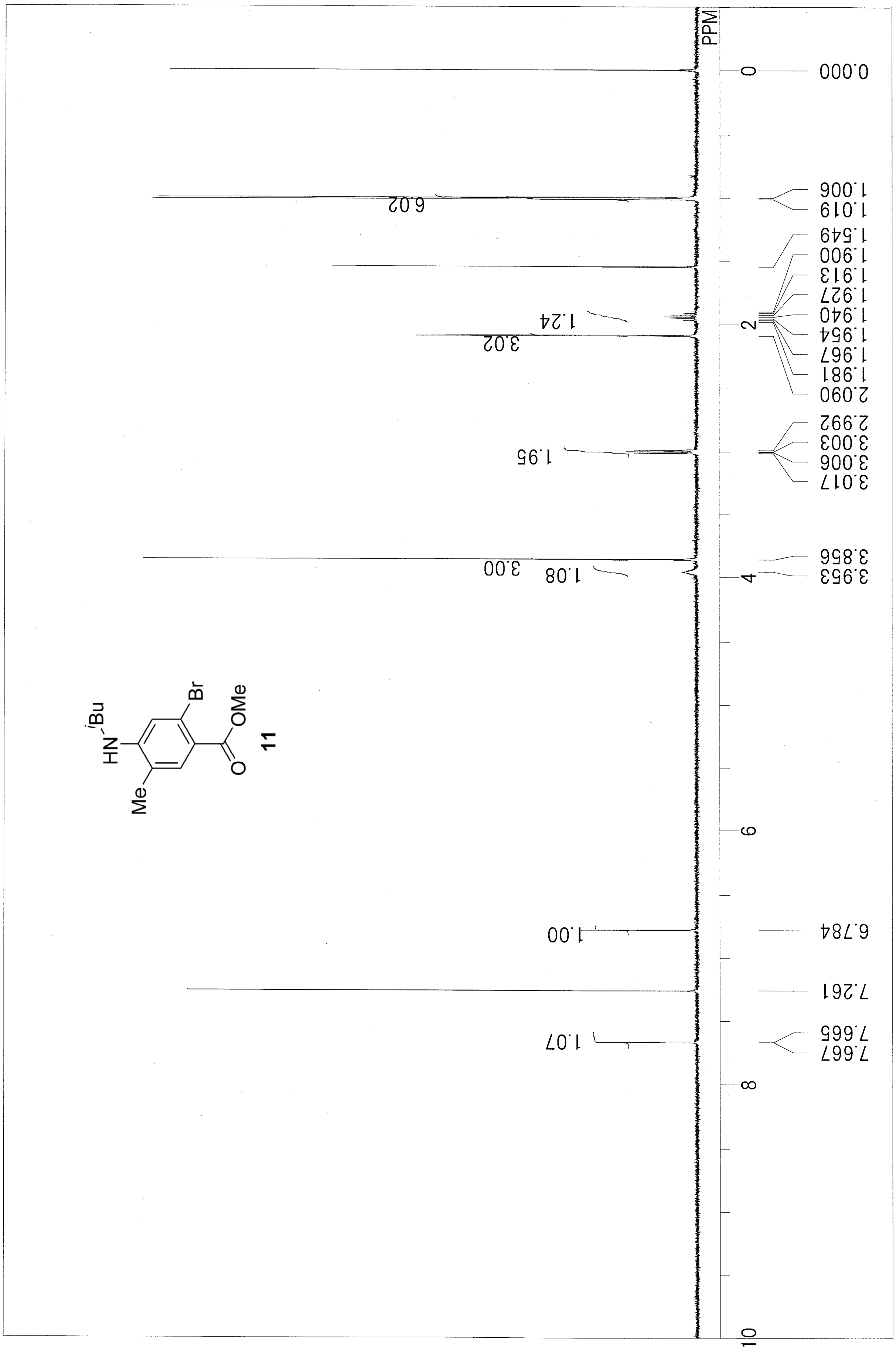

^{13}C NMR of **11** ($CDCl_3$, 126 MHz)

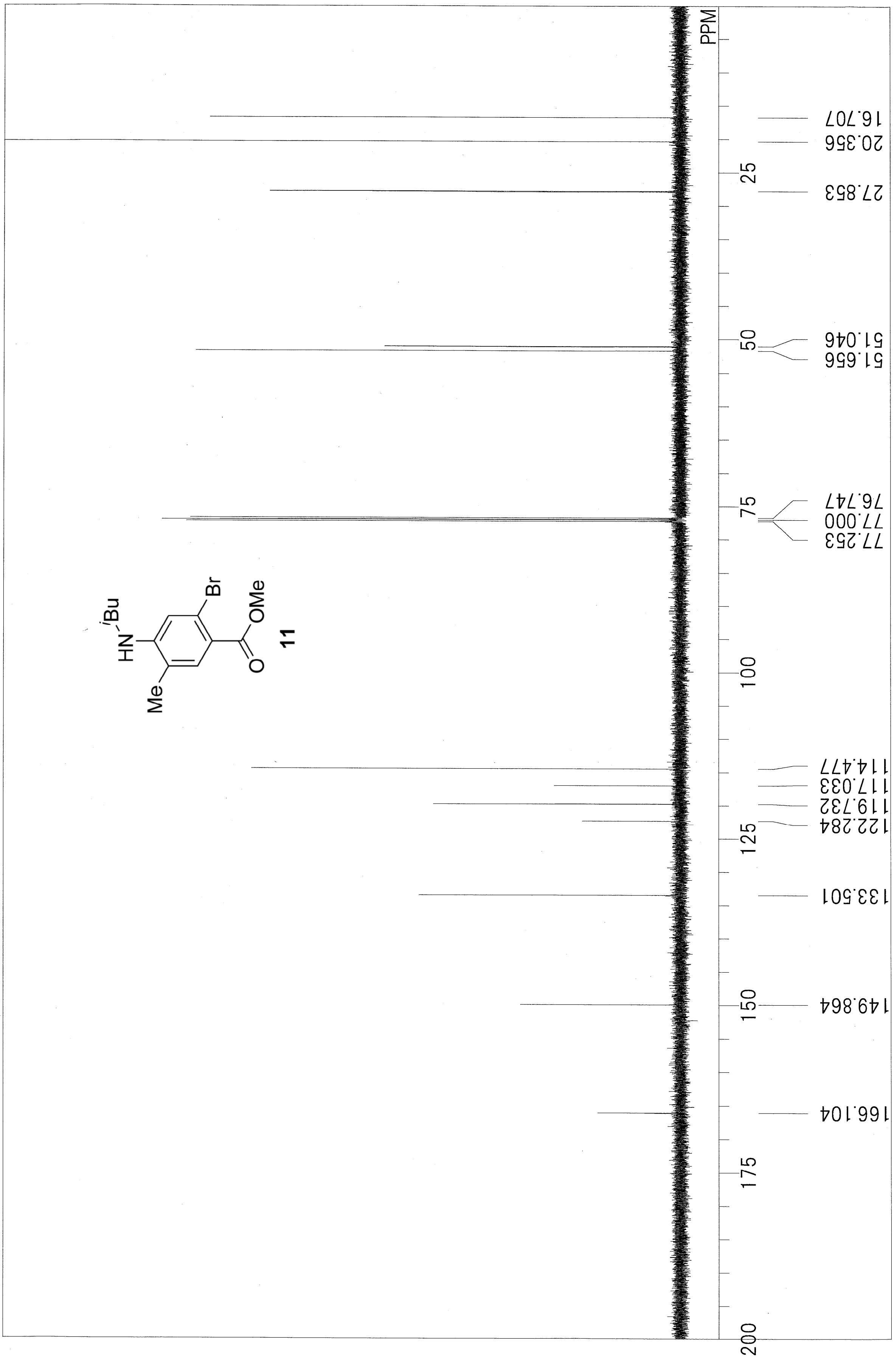

^{1}H NMR of **12** ($CDCl_3$, 500 MHz)

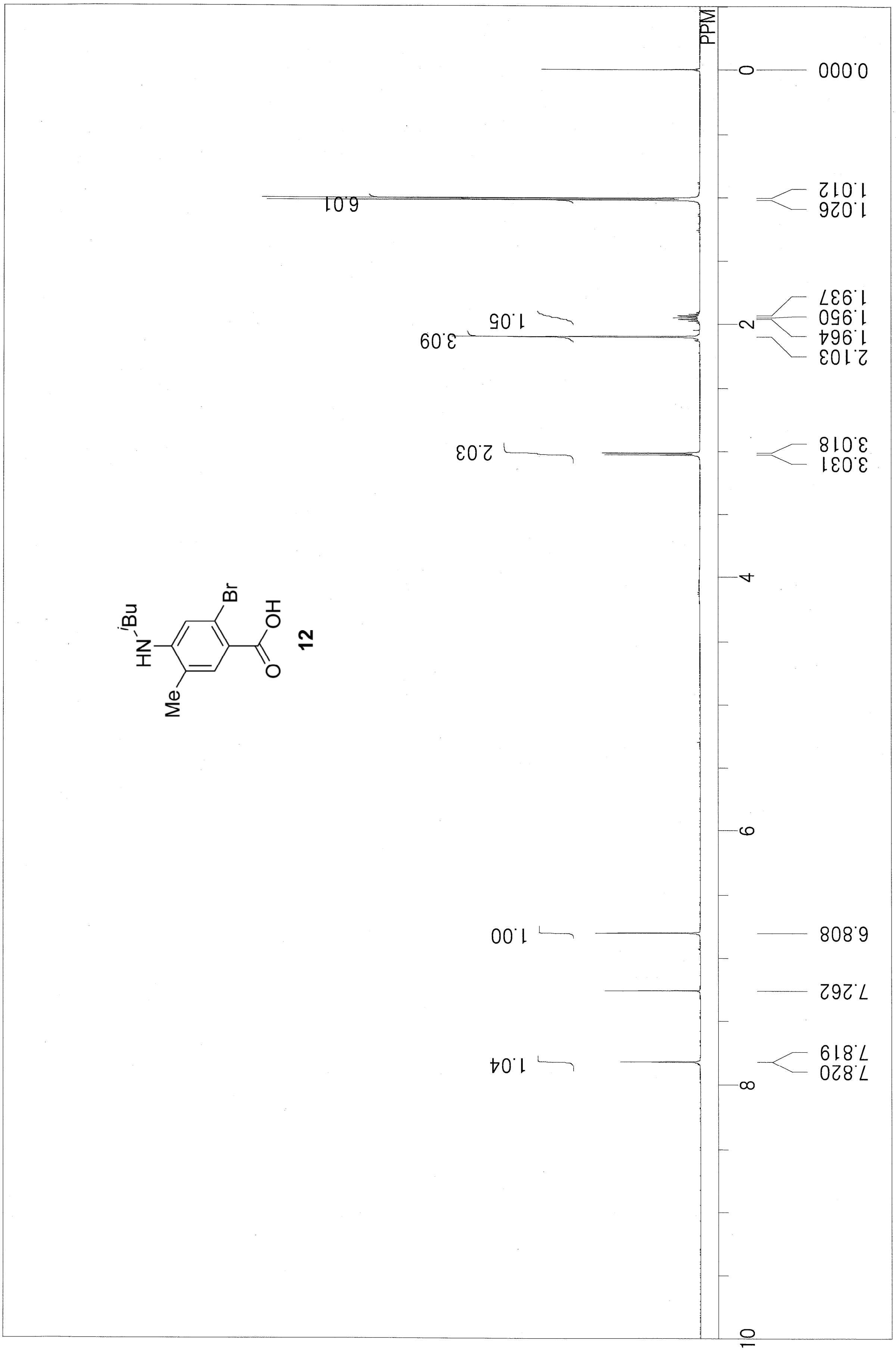

^{13}C NMR of **12** ($CDCl_3$, 126 MHz)

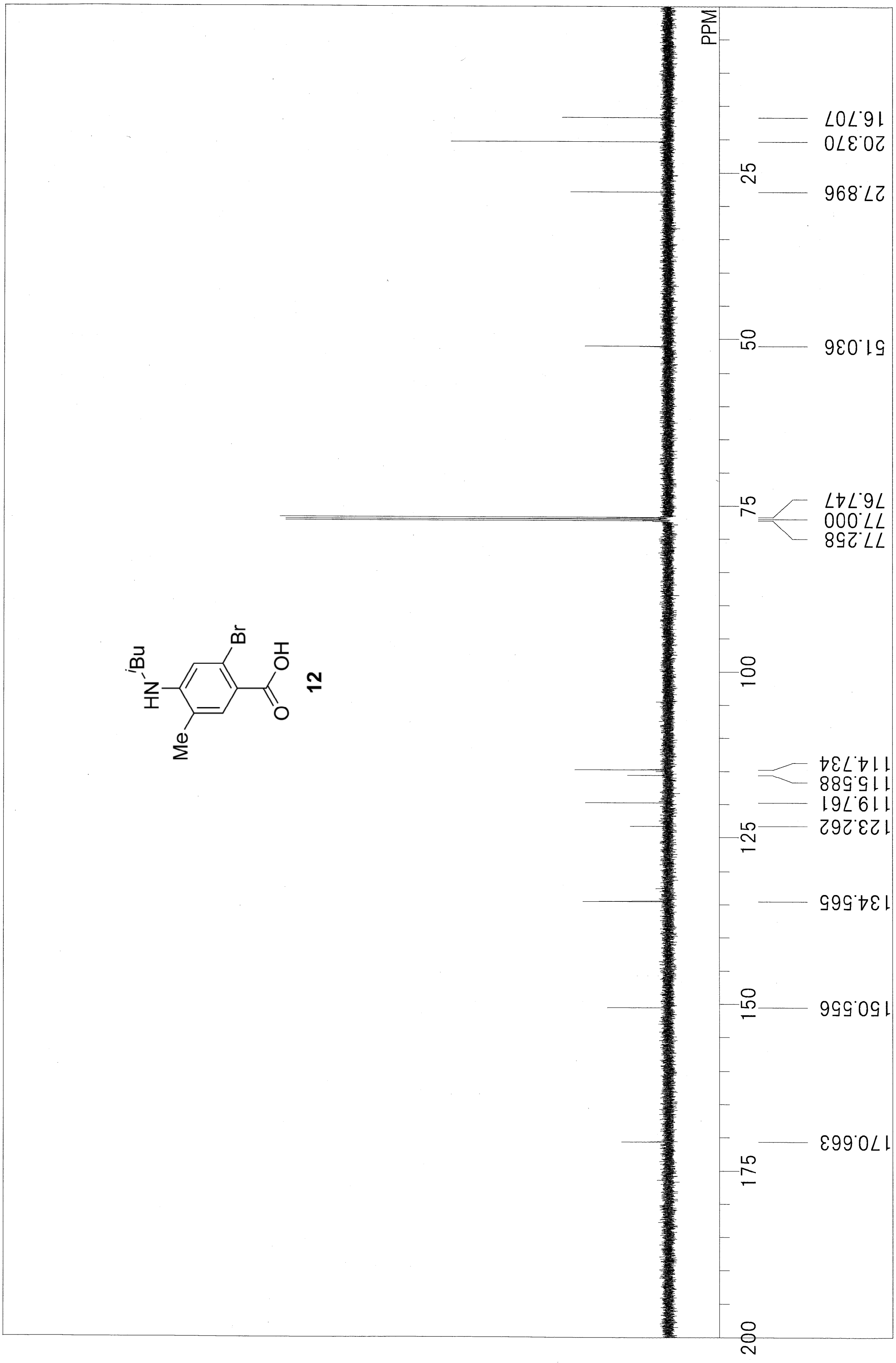

^1H NMR of *syn*-**6** ($CDCl_3$, 500 MHz)

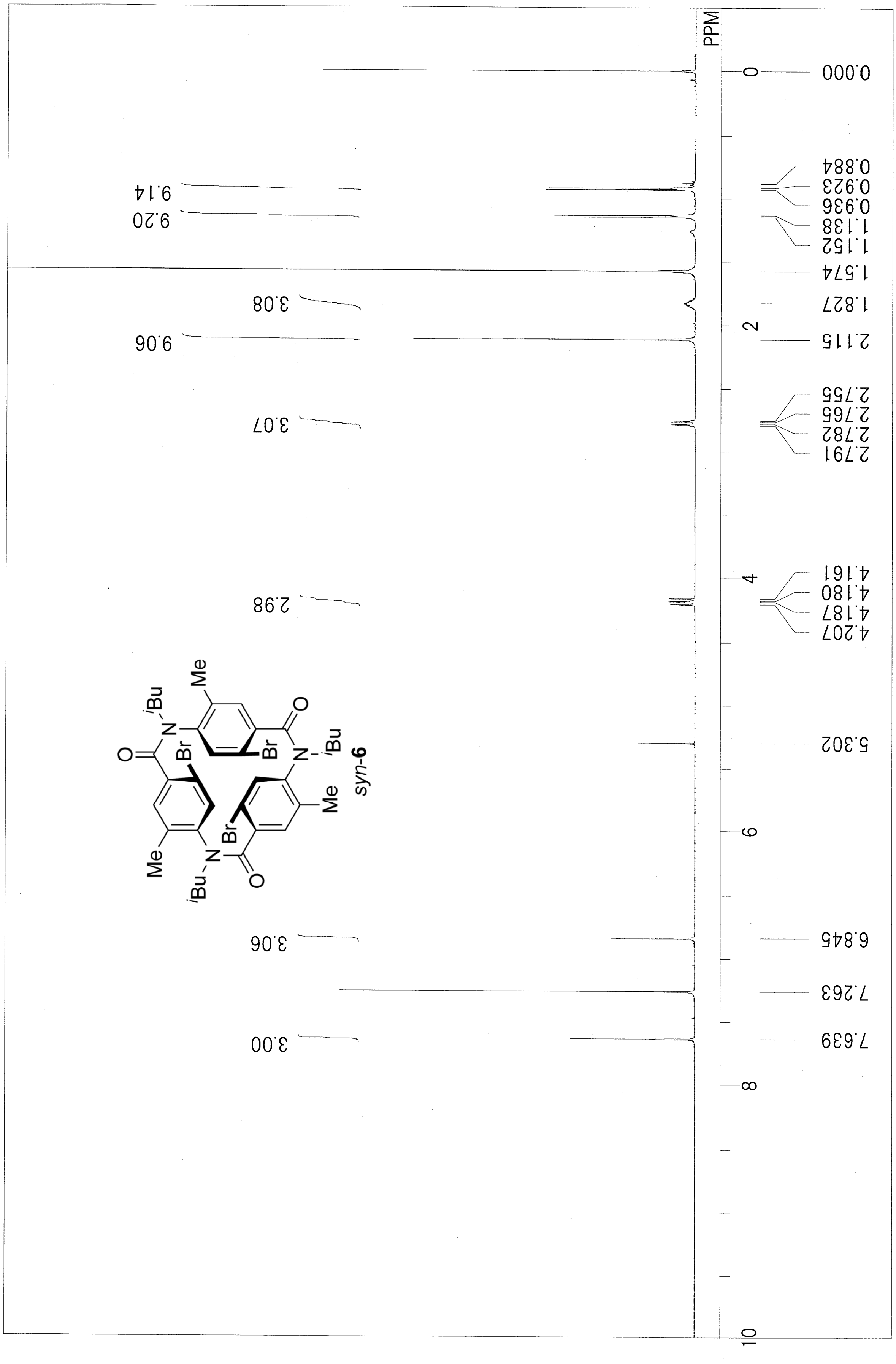

^{13}C NMR of *syn*-**6** ($CDCl_3$, 126 MHz)

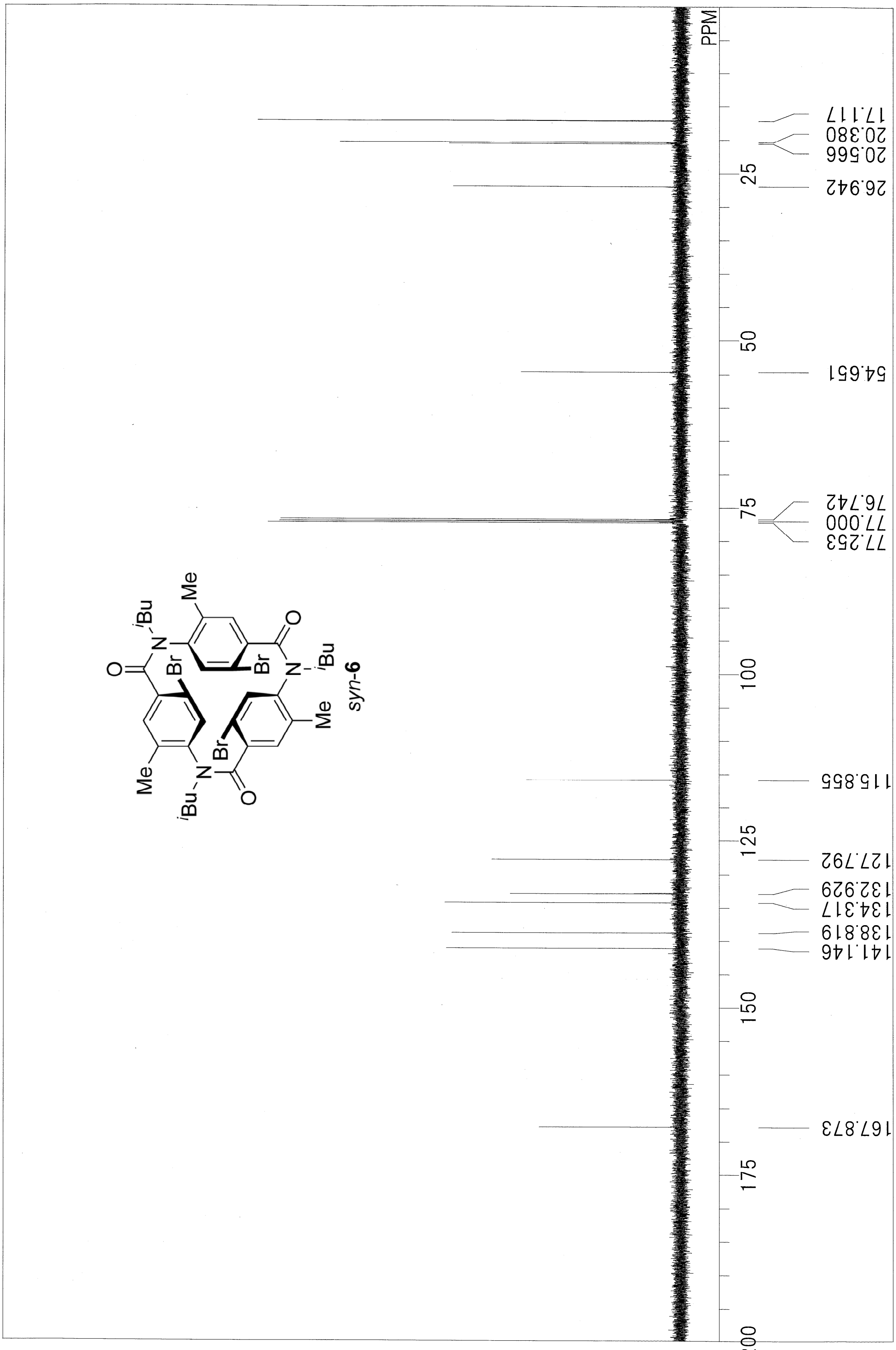

^{1}H NMR of *anti*-**6** ($CDCl_3$, 500 MHz)

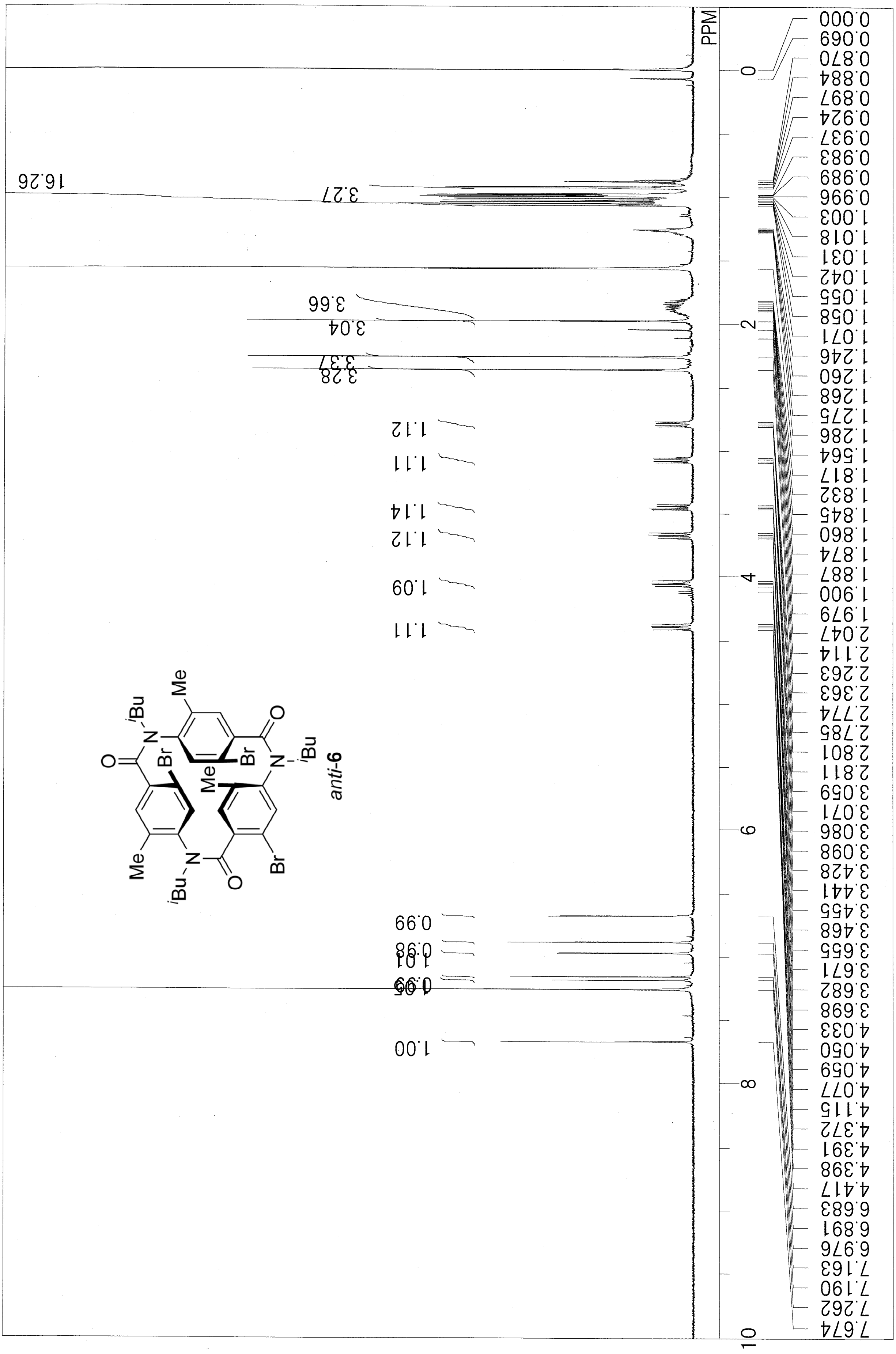

^{13}C NMR of *anti*-**6** ($CDCl_3$, 126 MHz)

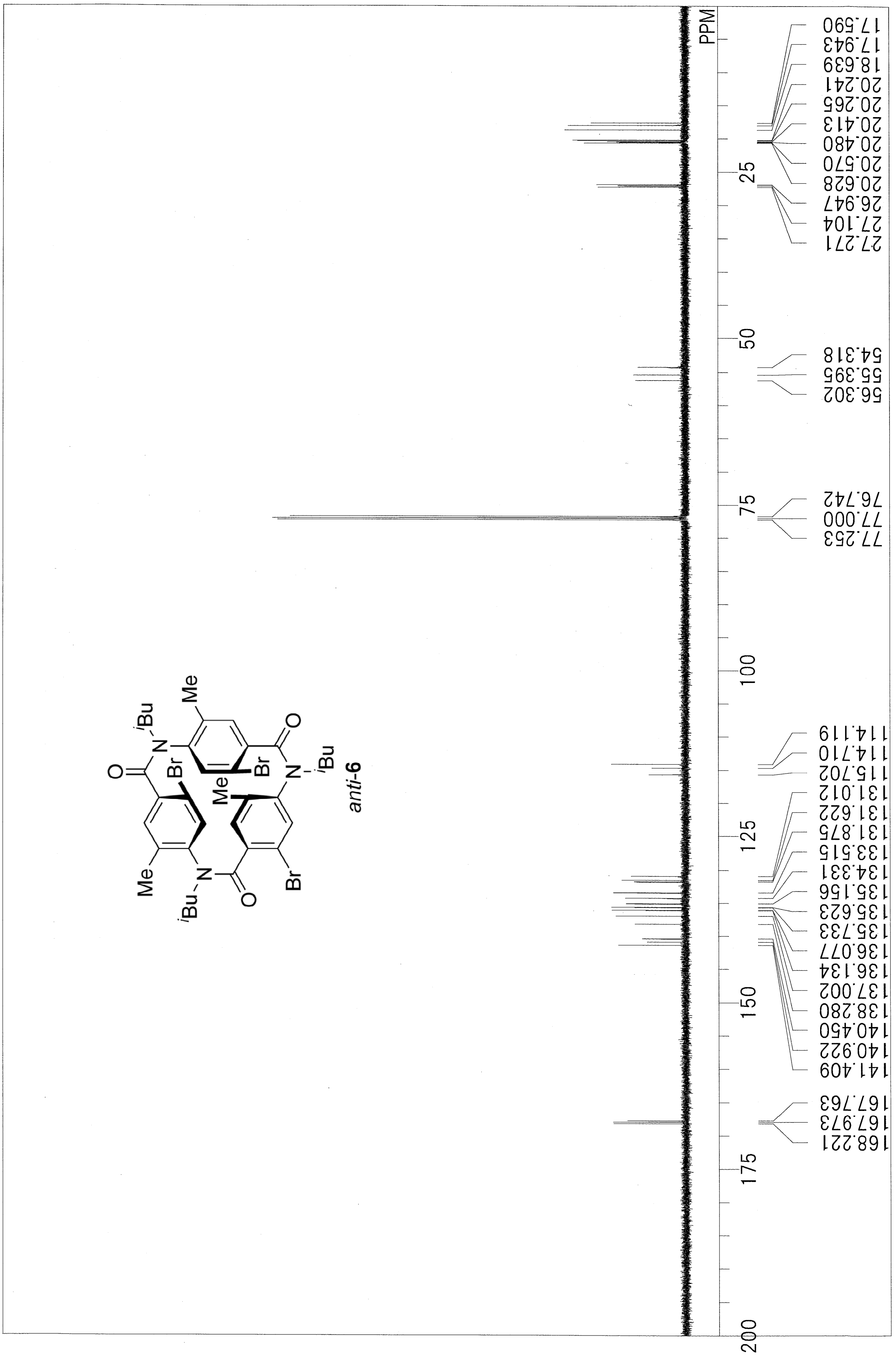

^{1}H NMR of **3** ($CDCl_3$, 500 MHz)

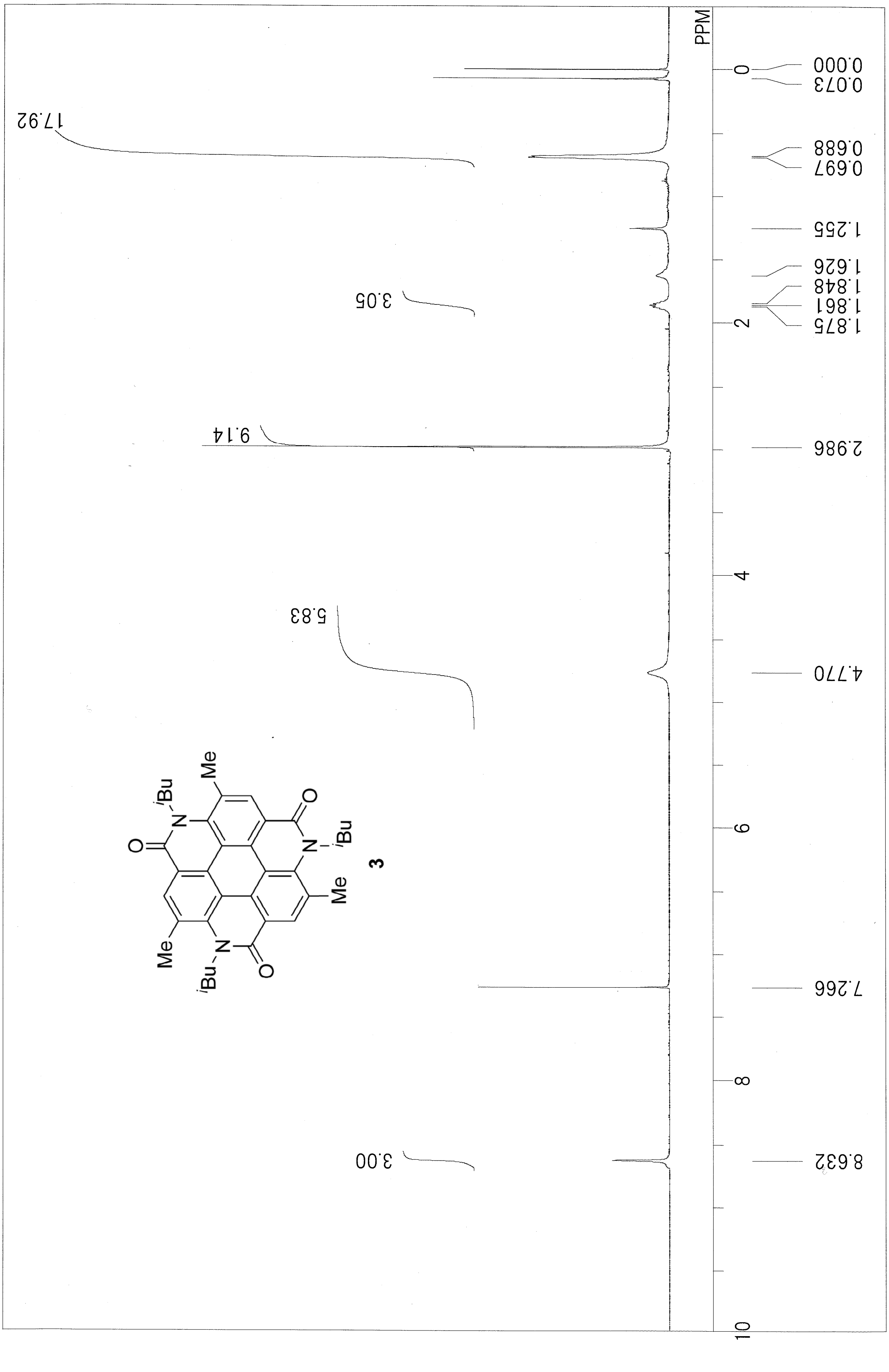

^{13}C NMR of **3** ($CDCl_3$, 126 MHz)

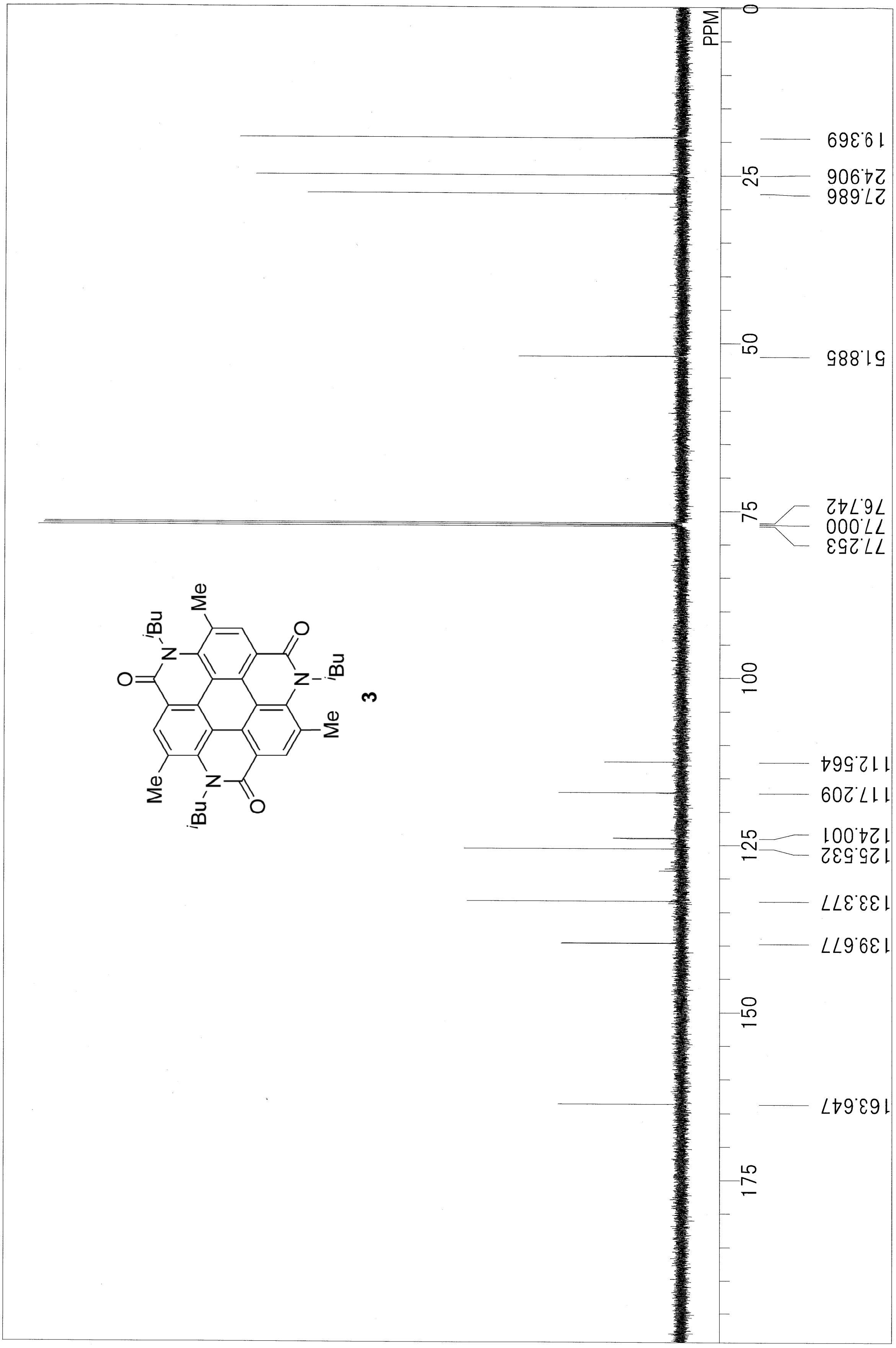

^1H NMR of **13** ($CDCl_3$, 500 MHz)

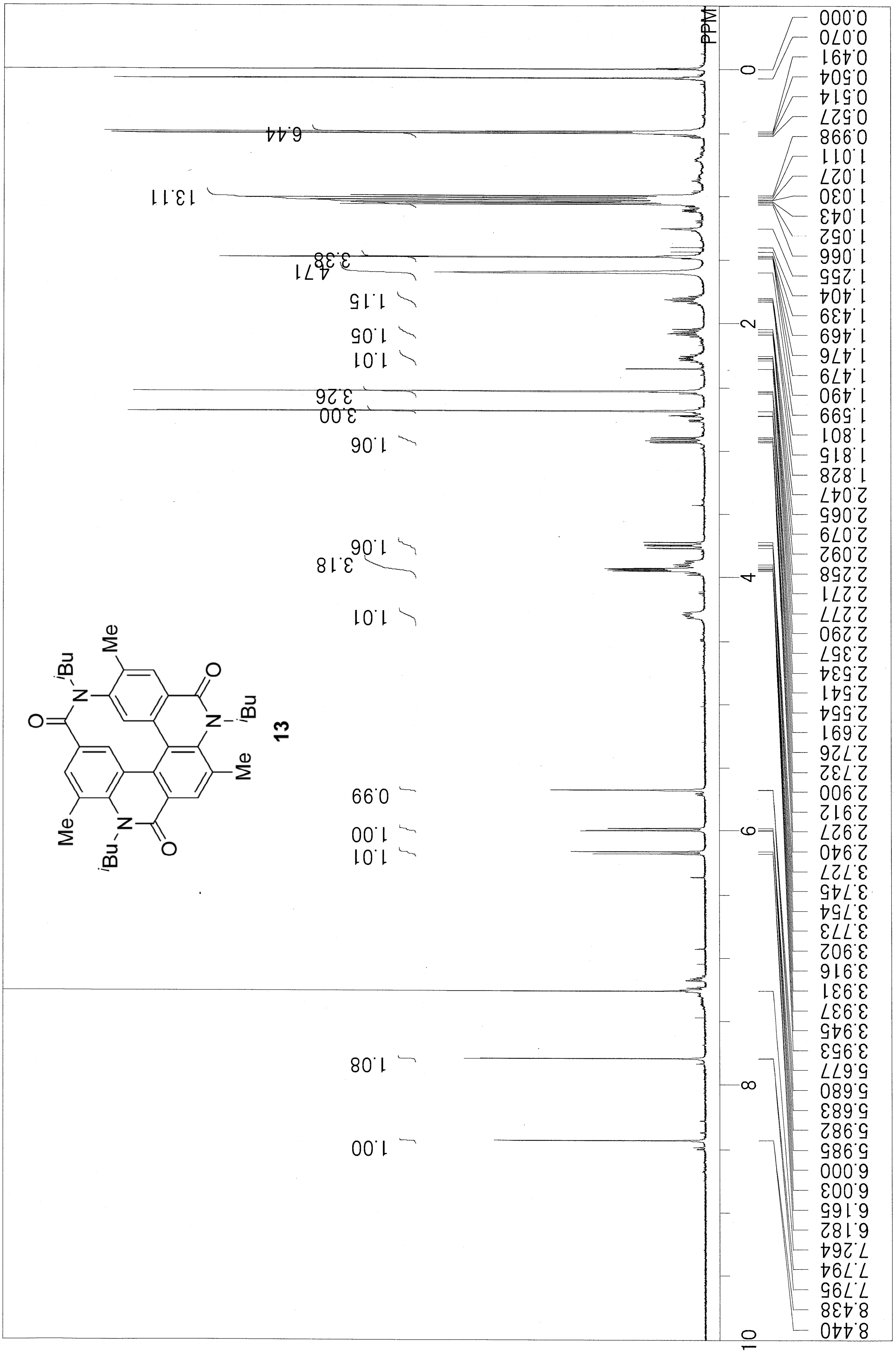

^{13}C NMR of **13** ($CDCl_3$, 126 MHz)

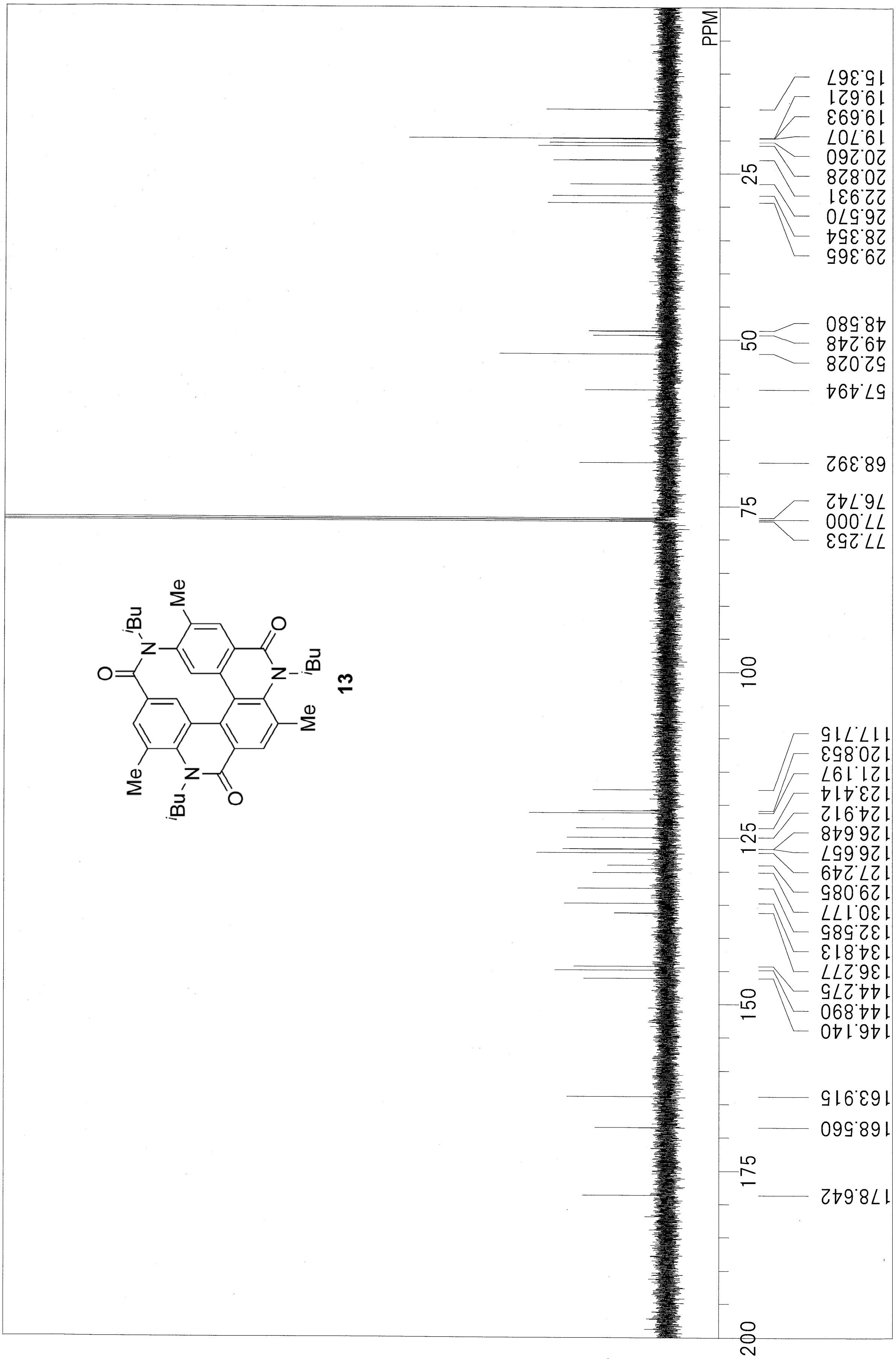

Supplement: RA-012-D2RA04035B-s001 [file RA-012-D2RA04035B-s001.pdf]
